# Taxonomic study on the photid amphipods (Senticaudata, Corophiida, Photoidea, Photidae) from Korean waters, with descriptions of a new genus and seven new species

**DOI:** 10.3897/zookeys.886.38511

**Published:** 2019-11-05

**Authors:** Tae Won Jung, Charles Oliver Coleman, Seong Myeong Yoon

**Affiliations:** 1 Research Center for Endangered Species, National Institute of Ecology, Yeongyang, 36531, South Korea National Institute of Ecology Yeongyang South Korea; 2 Museum für Naturkunde Berlin, Leibniz-Institute for Evolution and Biodiversity Science, 10115 Berlin, Germany Museum für Naturkunde Berlin Berlin Germany; 3 Department of Biology, Chosun University, Gwangju 61452, South Korea Chosun University Gwangju South Korea

**Keywords:** *Exiliphotis petila*, Korea, *Latigammaropsis careocavata*, lectotype, morphology, *Photis bronca*, *Photis longicaudata*, *Photis longicarpus*, *Photis posterolobus*, *Podoceropsis insinuomanus*, *Podoceropsis pseudoclavapes*, taxonomy

## Abstract

In this paper, seven new species of the family Photidae from Korean waters are described and illustrated in detail. Among them, *Exiliphotis petila***sp. nov.** is a monotype of the newly reported *Exiliphotis***gen. nov.**, which is characterized by slenderer and more elongate pereopods 5–7 than those of other genera of the family Photidae. The genus *Latigammaropsis* is reported for the first time from Korean waters based on the description of *Latigammaropsis careocavata***sp. nov.**, which is differentiated from other *Gammaropsi*s group by having weakly sexual dimorphic gnathopods 2, those have simple palmar margins in both sexes. Three new species of *Photis* are also described: *Photis bronca***sp. nov.**, *Photis posterolobus***sp. nov.**, and *Photis longicarpus***sp. nov.** The formerly misidentified *Photis longicaudata* from Japan and China as well as the Korean material could be classified as a new species, *P. bronca***sp. nov.** For that, the syntypes of *P. longicaudata* were re-examined, and lectotype and paralectotypes were newly designated in this study. *Photis bronca***sp. nov.** is characterized by quadrate tooth on the palmar margin medially on gnathopod 2 in both sexes. *Photis posterolobus***sp. nov.** shows a pointed posterior lobe on the ischium and a well-developed process of the propodus on male gnathopod 2. *Photis longicarpus***sp. nov.** can be distinguished from other species of the genus by very elongate carpus of male gnathopod 1. Two new species belonging to the genus *Podoceropsis* are also reported: *Podoceropsis insinuomanus***sp. nov.** has a strongly bisinuous palmar margin on male gnathopod 2, and *Podoceropsis pseudoclavapes***sp. nov.** differs from the closely related species of *Podoceropsis clavapes* by different shape of the palmar margin and shorter dactylus of male gnathopod 2. Additionally, a key to the Korean species of Photidae is provided.

## Introduction

The family Photidae was first proposed by Boeck (1871) as a subfamily Photinae, belonging to Gammaridea Latreille, 1802, to encompass the genera *Photis* Krøyer, 1842, *Microprotopus* Norman, 1866, and *Xenoclea* Boeck, 1870. The Photinae was raised to family level by Boeck (1872). He erected three subfamilies: the Leptocheirinae Boeck, 1970 with the genera *Leptocheirus* Zaddach, 1844 and *Goesia* Boeck, 1970; the Photinae with the genera *Photis*, *Microprotopus* and *Xenoclea*; and the Microdeutopinae Boeck, 1870 with the genera *Microdeutopus* Costa, 1853, *Aora* Krøyer, 1842, *Autonoe* Bruzelius, 1849, *Protomedeia* Krøyer, 1842, *Gammaropsis* Lilljeborg, 1854, and *Podoceropsis* Boeck, 1870 (Boeck 1872). Sars (1883) united the Leptocheirinae and Photinae into Photidae including Ptilocheirus (Leptocheirus), *Photis*, *Microprotopus*, and *Xenoclea*. Later, Stebbing (1888) rejected the subfamily concept and re-established the Photidae, including all genera of the previous subfamilies except for *Goesia* and *Xenoclea*. For a while, all taxa of the Photidae were included in the Isaeidae, which was originally conceived by Dana (1853) and later revised by Barnard (1969). However, a subsequent work by Barnard (1973) suggested a huge superfamily Corophioidea consisting of the families Ampithoidae, Biancolinidae, Cheluridae, Corophiidae, Ischyroceridae, and Podoceridae, where most of the genera related to the photids were being incorporated into the Corophiidae. Later, Barnard and Karaman (1991) insisted on the large group of Corophioidea, all members of which are sharing a fleshy telson, and the photids were still included into the Corophiidae. On the other hand, Bousfield (1973, 1978) had maintained the separation of the following families under the Corophioidea: Aoridae, Ampithoidae, Biancolinidae, Cheluridae, Corophiidae, Isaeidae, Ischyroceridae, Photidae, and Podoceridae. Myers and Lowry (2003) revised the higher-level classification for the suborder Corophiidea and suggested the new superfamily Photoidea consisting of Ischyroceridae, Kamakidae, and Photidae. At that time, the family Photidae included *Ampelisciphotis*, *Audulla*, *Dodophotis*, *Falcigammaropsis*, *Gammaropsis*, *Megamphopus*, *Microphotis*, *Papuaphotis*, *Photis*, and *Posophotis* (Myers and Lowry 2003). Recently, Lowry and Myers (2013) suggested the new suborder Senticaudata based on a cladistic analysis using morphological characters. The superfamily Photoidea is positioned under the parvorder Caprellidira. To date, the Photidae, under the Senticaudata, consists of 17 genera including more than 220 nominate species distributed worldwide (Jung et al. 2017).

In the Far East, the taxonomic studies on the Photidae have been carried out by Bulytscheva (1952), Gurjanova (1938, 1951, 1955), Hirayama (1984), Kudryashov and Tzvetkova (1975), Nagata (1961, 1965), and Ren (1992, 2000, 2006). Despite their species-richness, only five species belonging to three genera have been recorded from Korean waters: *Gammaropsis longipropodi* Hirayama, 1984; *Gammaropsis utinomii* (Nagata, 1961); *Photis stridulus* Jung et al., 2017; *Photis longicaudata* (Spence Bate & Westwood, 1862); and *Podoceropsis clavapes* Jung et al., 2017 (Kim et al. 2005; Kim and Lee 2010; Jung et al. 2017). Additionally, the specimens previously described under the name of *P. longicaudata* in the Far East (Nagata 1965; Hirayama 1984; Kim et al. 2005; Ren 2006) shows several differences to the original description and the specimens reported from the North Sea and the Mediterranean Sea (Bate and Westwood 1863; Sars 1894; Stebbing 1906), so it is doubtful that the material from the Far East belongs to the same species of the North Sea.

As a part of ongoing taxonomical studies on the photoid amphipods from Korean waters, seven new species are reported with diagnoses, detailed descriptions, and illustrations. A new monotypic genus *Exiliphotis* gen. nov. is established based on *Exiliphotis petila* sp. nov., and the genus *Latigammaropsis* Myers, 1995 is reported for the first time from Korean waters by the description of *Latigammaropsis careocavata* sp. nov. Three new species of the genus *Photis* Krøyer, 1842 are described: *Photis bronca* sp. nov., *Photis posterolobus* sp. nov., and *Photis longicarpus* sp. nov. Among them, *P. bronca* sp. nov. represents a revised species that has been misidentified as *P. longicaudata* in the Far East, including Japan, China, and Korea (Nagata 1965; Hirayama 1984; Kim et al. 2005; Ren 2006). Two new species belonging to the genus *Podoceropsis* Boeck, 1861 are also described: *Podoceropsis insinuomanus* sp. nov. and *Podoceropsis pseudoclavapes* sp. nov. Additionally, a key to Korean photid species is also provided.

## Materials and methods

Collected specimens were initially fixed in 80 % ethyl alcohol in the field and later preserved in 95 % ethyl alcohol after sorting in the laboratory. Specimens were stained with lignin pink. Their appendages were dissected under a stereomicroscope in petri dishes or excavated microscopic slides filled with glycerol using dissection forceps and needles (Leica M205). Specimens were mounted onto temporary slides using a glycerol-ethanol mixed solution. The illustrations made with a pencil under a light microscope (Leica DMLB) with the aid of a drawing tube. Later on, the drawings were scanned, digitally vectorized, and arranged in plates using the methods described by Coleman (2003, 2009). The examined material is deposited in the collection of the National Institute of Biological Resources (**NIBR**) of Korea. The syntype series of *Photis longicaudata* deposited in The Natural History Museum, London were examined for comparison to *Photis bronca* sp. nov. Further on, a lectotype and paralectotypes of *P. longicaudata* were designated based on the present study.

## Systematic accounts

### Order Amphipoda Latreille, 1816

**Suborder Senticaudata Lowry & Myers, 2013**

**Infraorder Corophiida Leach, 1814**

**Superfamily Photoidea Boeck, 1871**

#### Family Photidae Boeck, 1871

##### Genus Exiliphotis gen. nov.

Taxon classificationAnimaliaAmphipodaPhotidae

9303B708-0B6A-5F6C-8A87-DF30AB4724AB

http://zoobank.org/BD79F0AE-7A22-4BD8-9E3E-C46579A1AE67

###### Type species.

*Exiliphotis petila* sp. nov. (original designation by monotypy)

###### Etymology.

The composite epithet of the generic name, *Exiliphotis*, is a combination of the Latin word *exilis* and the generic name of *Photis*. This name refers to slender and elongate pereopods 5–7 with a noticeably narrower basis than those of other photid species. Gender is feminine.

###### Diagnosis.

Antenna 1 peduncle 2^nd^ article 2.0× as long as 1^st^ article; accessory flagellum uniarticulated, vestigial. Gnathopod 1 carpus broad, 2.0× as long as wide, 0.8× as long as basis, propodus as long and as wide as carpus, posterior margin more convex than anterior margin, free from carpal lobe proximally, palmar margin convex, not defined distinctively; dactylus elongate, 0.8× as long as propodus. Gnathopod 2 subequal to gnathopod 1; carpus 0.7× as long as basis and that of gnathopod 1; propodus as long as basis, 1.6× as long as carpus, posterior margin convex, not free from carpal lobe proximally, palmar margin oblique, strongly bisinuated; dactylus 0.7× as long as propodus, exceeding palm. Pereopod 5 coxa bilobed, as long and 1.5× as wide as that of pereopod 4, anterior lobe 0.9× as wide as basis; basis subovoid, 1.3× as wide as that of pereopod 4, half as wide as long; merus and carpus not lobed. Pereopod 6 similar to pereopod 5 except for coxa. Pereopod 7 1.3× as long as pereopod 6; basis subrectangular, as wide and 1.2× as long as that of pereopod 6; merus slender, 0.8× as long as basis, slightly lobed posteriodistally; carpus not lobate, 0.7× as long as merus; propodus 1.9× as long as carpus. Pleonal epimera 2 and 3 subrectangular, rounded ventrally. Uropod 1 peduncle without distoventral spine; both rami 0.6× as long as peduncle, terminated by rounded apex bearing robust seta. Uropod 2 0.7× as long as uropod 1; both rami 0.9× as long as peduncle, terminated by rounded apex bearing robust seta. Uropod 3 0.7× as long as uropod 2, inner ramus half as long as outer ramus, diminished distally; outer ramus as long as peduncle, biarticulated, 2^nd^ article vestigial, with 2 elongate setae subapically. Telson trapezoidal in dorsal view, 2.1× as wide as long, distal margin not acute, with 1 terminal spine on each side.

###### Remarks.

*Exiliphotis* gen. nov. is similar to *Graciliphotis* Myers, 2009 in having relatively short coxae, which differs from those of other photids (Myers 2009). However, *Exiliphotis* gen. nov. notably differs from *Graciliphotis* by the following differences: gnathopods 1 and 2 carpal lobes are slightly shorter and stouter; pereopods 5–7 are almost linear and elongate, particularly their basis are narrowly expanded; uropods 1 and 2 rami are terminated by the rounded apex bearing one small robust seta; uropod 3 is about half as long as in *Graciliphotis*, 0.7 times as long as uropod 2; uropod 3 outer ramus is biarticulated (uniarticulated in *Graciliphotis*); and uropod 3 inner ramus is half as long as the outer ramus, while it is vestigial in *Graciliphotis* (Myers 2009).

##### Exiliphotis petila sp. nov.

Taxon classificationAnimaliaAmphipodaPhotidae

7D8E8F46-A67D-5B61-B3B0-FF5C42B81FB6

http://zoobank.org/FF232410-A5DE-41CD-B040-0FEF54FA5FC1

Figs 1 , 2 , 3

###### Etymology.

The epithet of the specific name, *petila*, is originated from the Latin word *petilus*. This name refers to the shape of pereopod 5 coxa and basis, and pereopods 6 and 7 basis, which are slenderer than those of other photid amphipods.

###### Material examined.

Holotype: ♂ (3.7 mm), NIBRIV0000806532. Daegwantal-do Island, Jeju-do, South Korea (33°43'38"N, 126°20'13"E), 21 Nov 2012, grab sampler (about 36 m depth), by Prof. HY Soh.

###### Description.

**Figure 1.**
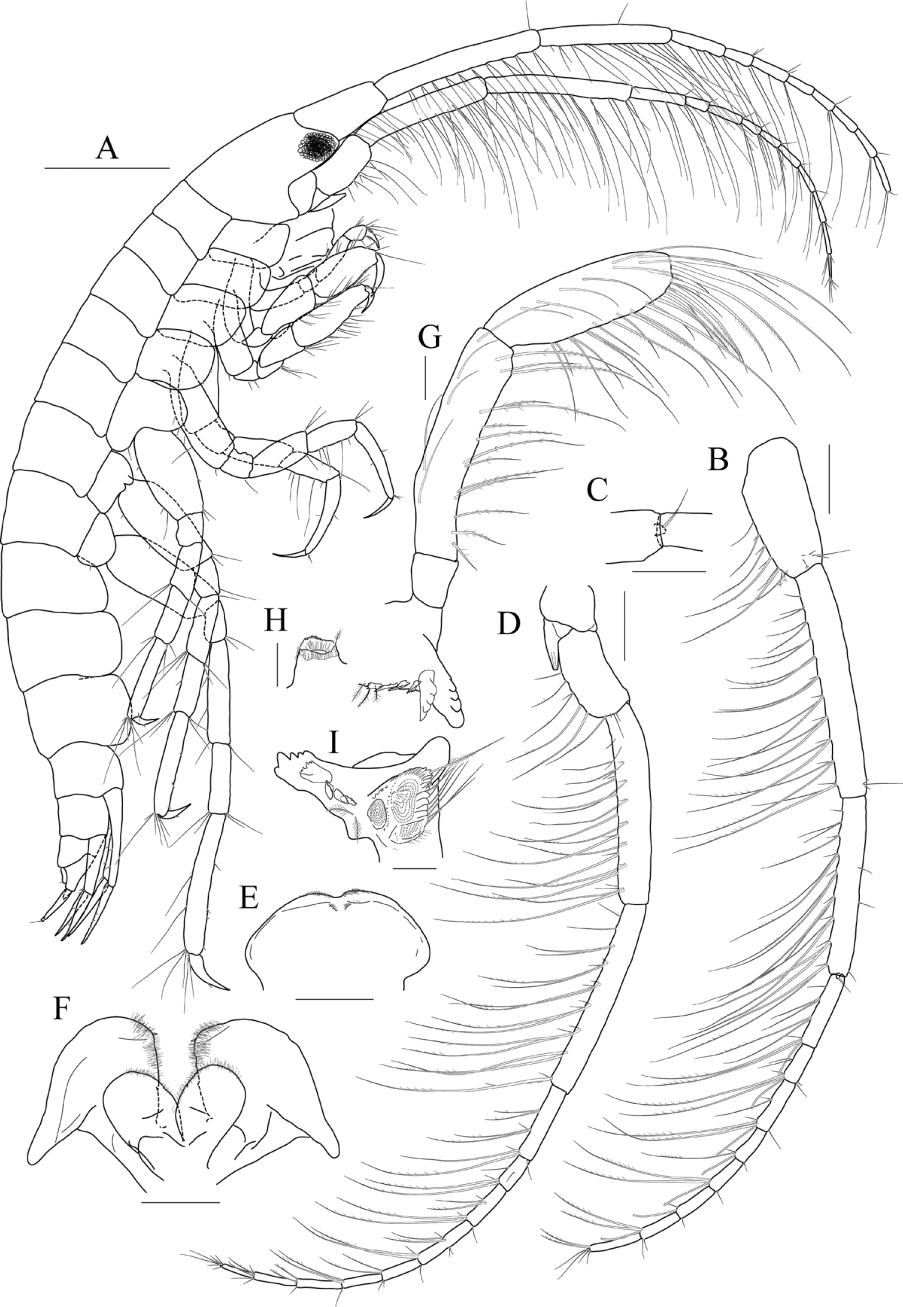
*Exiliphotis petila* sp. nov., holotype, NIBRIV0000806532, male, 3.7 mm, Daegwantal-do Island, South Korea. **A** habitus **B, C** antenna 1 **D** antenna 2 **E** upper lip **F** lower lip **G, H** left mandible **I** right mandible. Scale bars: 0.05 mm (**G–I**), 0.1 mm (**C, E, F**), 0.2 mm (**B, D**), 0.5 mm (**A**).

***Holotype male.*** Head (Fig. 1A) 0.9× as long as pereonites 1–3 combined; lateral cephalic lobe produced anteriorly, apex rounded; eye circular, large, occupying most of lateral cephalic lobe; antennal sinus deep.

Antenna 1 (Fig. 1B, C) 0.7× as long as body; peduncle 1^st^ article stout, 0.7× as long as head; without robust seta posteriodistally; 2^nd^ article 2.0× as long as 1^st^ article; 3^rd^ article 0.8× as long as 2^nd^ article; accessory flagellum uniarticulated, vestigial; flagellum 2.0× as long as peduncle 3^rd^ article, composed of nine articles (terminal article rudimentary).

Antenna 2 (Fig. 1D) 1.1× as long as antenna 1; peduncle 4^th^ article 0.7× as long as 2^nd^ article of antenna 1; 5^th^ article as long as 4^th^ article; flagellum 1.9× as long as peduncle 5^th^ article, composed of ten articles (terminal article rudimentary).

Upper lip (Fig. 1E) convex anteriorly, with notch in the middle, covered with minute setae.

Lower lip (Fig. 1F) inner lobe subovoid, outer lobe apex rounded, covered with minute setae; mandibular process well developed.

Mandibles (Fig. 1G–I) with 1/2 and 4-dentate incisor, 4-dentate lacinia mobilis, and four raker setae on left mandible; with 5-dentate incisor, minutely dentate lacinia mobilis, and three raker setae on right mandible; molar well developed, triturative, with seven setae along the distal margin of right mandible; palp asymmetrical, composed of three articles, 3^rd^ article distally rounded, 0.8× as long as 2^nd^ article, with setae extending along most of posteriodistal margin.

**Figure 2:**
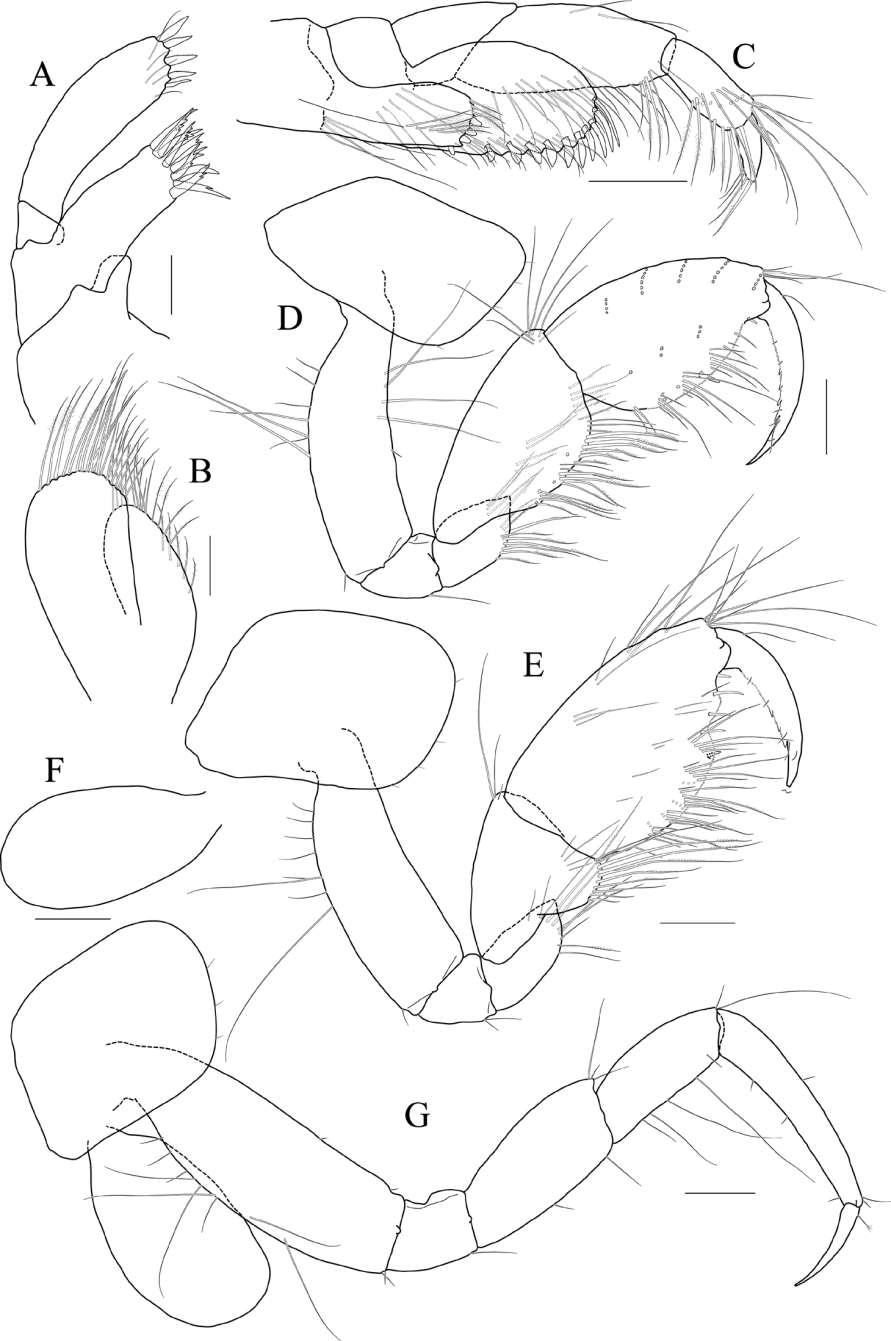
*Exiliphotis petila* sp. nov., holotype, NIBRIV0000806532, male, 3.7 mm, Daegwantal-do Island, South Korea. **A** maxilla 1 **B** maxilla 2 **C** maxilliped **D** gnathopod 1 **E, F** gnathopod 2 **G** pereopod 3. Scale bars: 0.05 mm (**A, B**), 0.1 mm (**C–G**).

Maxilla 1 (Fig. 2A) inner lobe small, without seta; outer lobe with ten dentate setae on apical margin; palp biarticulated, 2^nd^ article slightly dilated distally, with five dentate setae apically and five simple setae subapically.

Maxilla 2 (Fig. 2B) inner lobe with an oblique row of five weakly plumose setae on surface; outer lobe lager than inner lobe, expanded distally.

Maxilliped (Fig. 2C) inner lobe subrectangular, with three nodular setae apically and one medial nodular seta subdistally; outer lobe semiovoid, apex beyond half of 2^nd^ palp article, lined with ten stout setae on medial to apical margins; palp composed of four articles, 3^rd^ article a little expanded distally, half as long as 2^nd^ article, 4^th^ article 0.6× as long as 3^rd^ article, with elongate seta apically (1.1× as long as 4^th^ article).

Gnathopod 1 (Fig. 2D) coxa subtrapezoidal, 0.7× as wide as long, slightly produced anterioventrally; basis subtrapezoidal, anterior margin distal lobe weakly developed, with four elongate setae in the middle, posterior margin convex subproximally, with seven setae; carpus broad, 2.0× as long as wide, 0.8× as long as basis, with carpal lobe broad and blunt; propodus as long and as wide as carpus, anterior margin evenly rounded, with five oblique rows of elongate setae submarginally, posterior margin more convex than anterior margin, free form carpal lobe proximally, palmar margin convex, finely serrated on distal 3/4 margin, not defined distinctively, with one robust seta medially at 1/3 length distally; dactylus elongate, 0.8× as long as propodus, inner margin finely serrated, with four teeth.

Gnathopod 2 (Fig. 2E, F) subequal to gnathopod 1; coxa subtrapezoidal, 1.1× as wide as that of gnathopod 1, 0.7× as wide as long, rounded ventrally, not produced anterioventrally; coxal gill subovoid, 0.9× as long as coxa; basis similar to that of gnathopod 1, without setae on anterior margin; carpus 0.7× as long as basis and that of gnathopod 1, carpal lobe also shorter than that of gnathopod 1, blunt posteriorly; propodus as long as basis, 1.6× as long as carpus, anterior margin evenly rounded, posterior margin convex, not free from carpal lobe proximally, finely serrated on distal half margin, palmar margin oblique, strongly bisinuated, finely serrated, defined by one robust seta medially; dactylus 0.7× as long as propodus, exceeding palm, inner margin finely serrated, with three teeth.

Pereopod 3 (Fig. 2G) coxa as wide but 0.9× as long as that of gnathopod 2, rounded ventrally, weakly produced anterioventrally, coxal gill subovoid, 1.1× as long as that of gnathopod 2; basis 1.4× as long as coxa, 0.3× as wide as long, posterior margin convex, with elongate setae; merus 0.6× as long as basis, 0.5× as wide as long, slightly lobate anteriodistally; carpus subrectangular, 0.9× as long as merus, slightly convex anteriorly; propodus tapering distally, 0.8× as long as basis; dactylus 0.4× as long as propodus.

**Figure 3:**
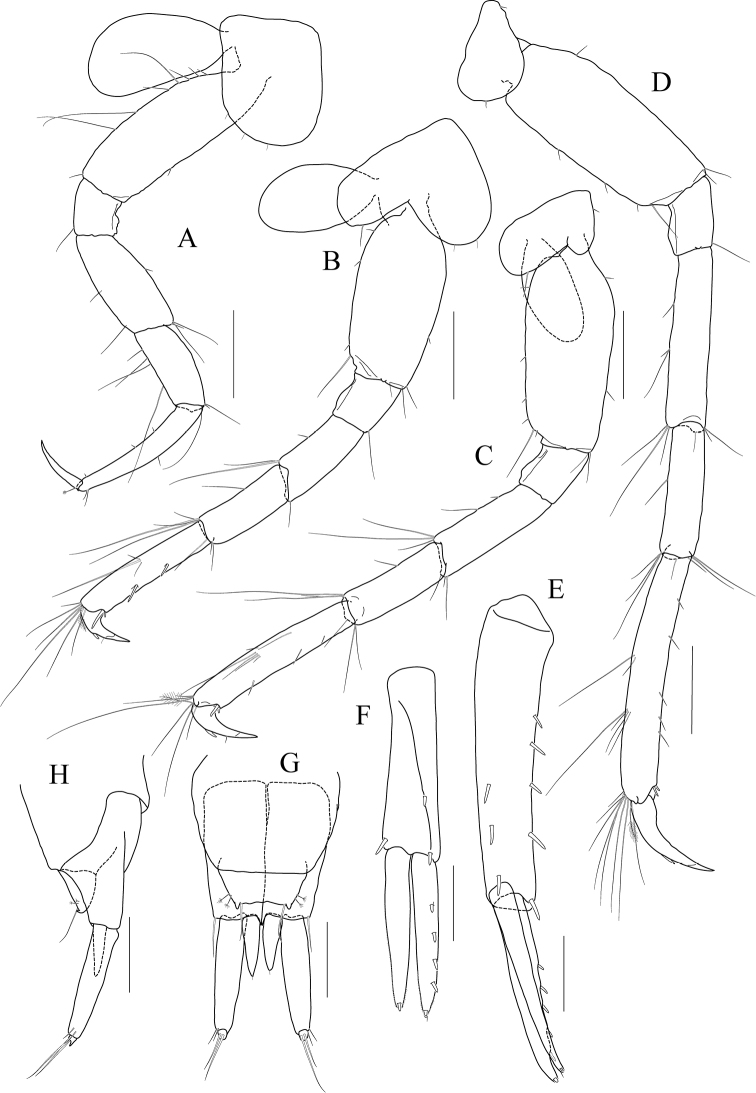
*Exiliphotis petila* sp. nov., holotype, NIBRIV0000806532, male. 3.7 mm, Daegwantal-do Island, South Korea. **A** pereopod 4 **B** pereopod 5 **C** pereopod 6 **D** pereopod 7 **E** uropod 1 **F** uropod 2 **G, H** uropod 3 and telson, dorsal (**G**) and lateral (**H**). Scale bars: 0.1 mm (**E–H**), 0.2 mm (**A–D**).

Pereopod 4 (Fig. 3A) similar to pereopod 3.

Pereopod 5 (Fig. 3B) slender; coxa bilobed, as long and 1.5× as wide as that of pereopod 4, anterior lobe expanded downward, 0.9× as wide as basis, rounded ventrally, posterior lobe expanded backwards, slightly smaller than anterior lobe; basis subovoid, 1.3× as wide as that of pereopod 4, half as wide as long, slightly wider proximally, posterior margin with one elongate seta and notched distally; merus and carpus not lobate, half as long as basis, with elongate setae at posteriodistal corners; propodus 1.5× as long as carpus, anterior margin with two robust setae and one pair of locking setae unequal in length; dactylus 0.4× as long as propodus, armed with one accessory cusp on outer margin.

Pereopod 6 (Fig. 3C) subequal to pereopod 5 except for coxa; coxa bilobed, anterior lobe small, posterior lobe as large as that of pereopod 4; basis subrectangular, as long and wide as that of pereopod 5, slightly wider proximally; merus and carpus not lobate, each 0.6× and 0.9× as long as basis, with elongate setae at posteriodistal corners; propodus 1.6× as long as carpus, anterior margin with simple setae and a pair of locking setae unequal in length; dactylus 0.4× as long as propodus, armed with one accessory cusp on outer margin.

Pereopod 7 (Fig. 3D) 1.3× as long as pereopod 6, slender; coxa unilobed, rounded ventrally, slightly dilated posterioventrally; basis subrectangular, as wide and 1.2× as long as that of pereopod 6, 0.4× as wide as long; merus slender, 0.8× as long as basis slightly lobate posteriodistally; carpus not lobate, 0.7× as long as merus; propodus 1.9× as long as carpus, slightly curved, anterior margin with simple setae and a pair of locking setae unequal in length; dactylus 0.4× as long as propodus, armed with one accessory cusp on outer margin.

Epimera 2–3 (Fig. 1A) subrectangular, rounded ventrally.

Uropod 1 (Fig. 3E) peduncle without distoventral spine, with five dorsolateral and three dorsomedial robust setae; outer ramus 0.6× as long as peduncle, with five dorsolateral robust setae, terminated by rounded apex bearing one robust seta; inner ramus as long as outer ramus, without marginal setae, terminated by rounded apex bearing one robust and one simple seta.

Uropod 2 (Fig. 3F) 0.7× as long as uropod 1; peduncle without distoventral spine, 0.6× as long as that of uropod 1, with one dorsomedial and two dorsolateral robust setae; outer ramus 0.9× as long as peduncle, with four dorsolateral robust setae, terminated by rounded apex bearing one robust and one simple seta; inner ramus as long as outer ramus, without marginal setae, terminated by rounded apex bearing one robust seta.

Uropod 3 (Fig. 3G, H) 0.7× as long as uropod 2; peduncle 0.7× as long as that of uropod 2; inner ramus half as long as outer ramus, diminished distally, with minute apical seta; outer ramus as long as peduncle, biarticulated, 2^nd^ article vestigial, with two elongate setae subapically.

Telson (Fig. 3G, H) trapezoidal in dorsal view, 2.1× as wide as long, distal margin not acute, with one terminal spine, one simple seta, and two sensory setae on each side.

###### Remarks.

See the remarks section under the genus.

##### Genus *Latigammaropsis* Myers, 1995

Korean name: Jjalb-eun-but-eun-ggo-ri-yeop-sae-u-sok

###### Latigammaropsis careocavata sp. nov.

Taxon classificationAnimaliaAmphipodaPhotidae

442A0A55-02F8-5F0E-B90D-49C6C4820E4B

http://zoobank.org/69BD8FB5-D648-463A-958D-830CC7A423FF

Figs 4 , 5 , 6 , 7

####### Etymology.

The composite epithet of the specific name, *careocavata*, is a combination of the Latin words *careo* and *cavatus*, meaning lacking excavation. This name refers to the shape of the palmar margin of gnathopod 2 in both sexes.

####### Material examined.

Holotype: ♂ (7.5 mm), NIBRIV0000806529. Sogueulbi-do Island, Gyeongsangnam-do, South Korea (34°34.571'N, 128°32.566'E), 7 May 2012, grab sampler (about 60 m depth), by Prof. HY Soh. Paratypes: 2 ♂♂ (4.5 and 4.8 mm), 3 ♀♀ (5.5–6.7 mm), NIBRIV0000848929. Same data as holotype.

####### Diagnosis.

Gnathopod 2 stout in both sexes (similar in shape), but basis and propodus less setose anteriorly in females; propodus palmar margin oblique, slightly convex, serrated, without excavations, defined by a single stout spine and defining seta elongate, longer in males.

####### Description.

**Figure 4:**
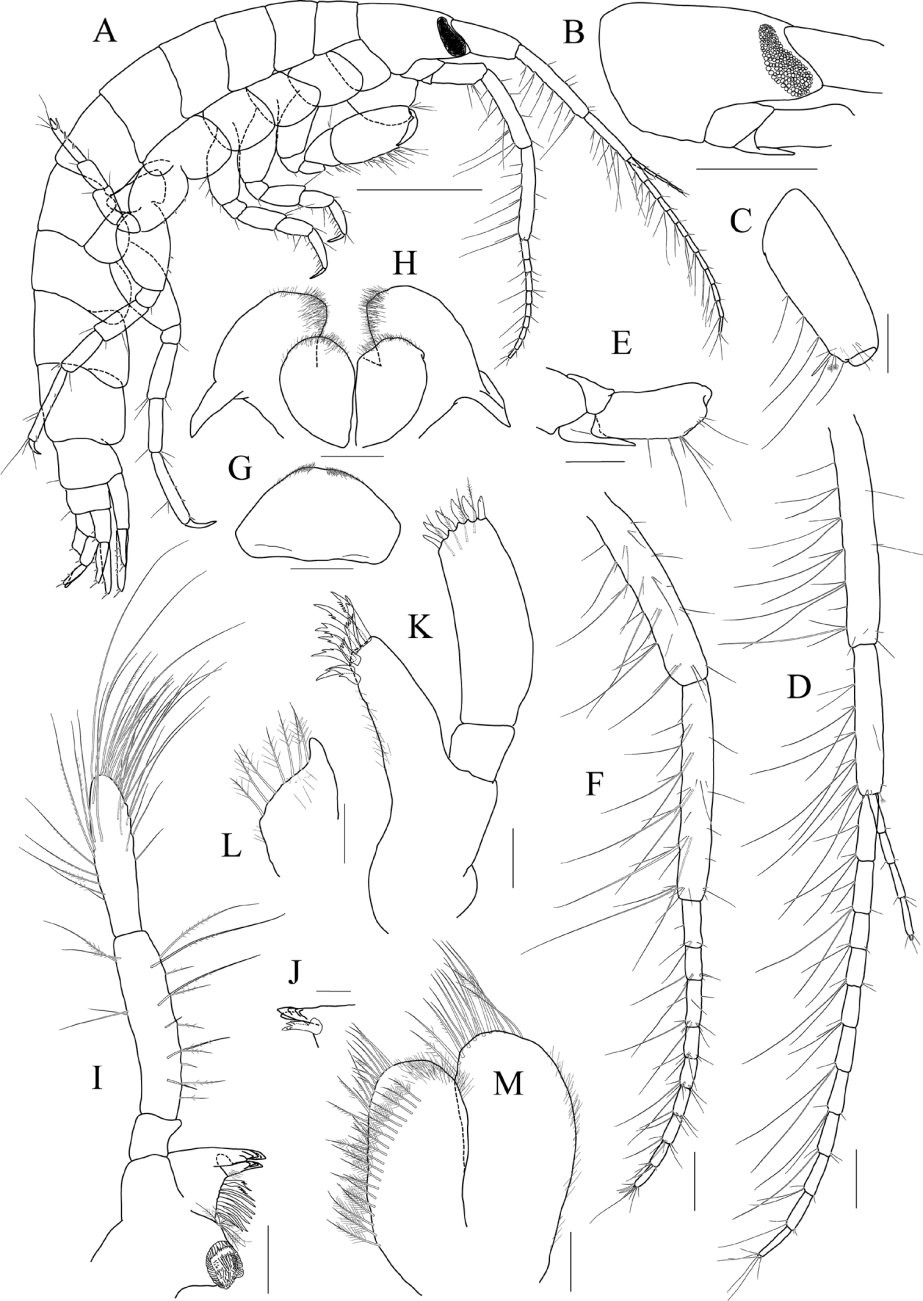
*Latigammaropsis careocavata* sp. nov., holotype, NIBRIV0000806529, male, 7.5 mm, Sogueulbi-do Island, South Korea. **A** habitus **B** head **C, D** antenna 1 **E, F** antenna 2 **G** upper lip **H** lower lip **I** left mandible **J** incisor and lacinia mobilis of right mandible **K, L** maxilla 1 **M** maxilla 2. Scale bars: 0.05 mm (**J–M**), 0.1 mm (**G–I**), 0.2 mm (**C–F**), 0.5 mm (**B**), 1.0 mm (**A**).

***Holotype male.*** Head (Fig. 4B) lateral cephalic lobe weakly produced anteriorly; eye lageniform, large; antennal sinus deep.

Antenna 1 (Fig. 4C, D) 0.6× as long as body; peduncle 1^st^ article stout, 0.7× as long as head, with one robust seta posteriodistally; 2^nd^ article 1.3× as long as 1^st^ article; 3^rd^ article 0.7× as long as 2^nd^ article; accessory flagellum as long as peduncle 3^rd^ article, composed of five articles (terminal article rudimentary); flagellum 0.9× as long as peduncle 1^st^–3^rd^ articles combined, composed of twelve articles (terminal article rudimentary).

Antenna 2 (Fig. 4E, F) as long as antenna 1; peduncle 4^th^, 5^th^ articles 0.9× as long as 2^nd^ article of antenna 1; flagellum 1.4× as long as peduncle 5^th^ article, composed of ten articles (terminal article rudimentary).

Upper lip (Fig. 4G) convex anteriorly, covered with minute setae.

Lower lip (Fig. 4H) inner lobe subovoid, outer lobe apex rounded, covered with minute setae; mandibular process well developed.

Mandibles (Fig. 4I, J) with 4-dentate incisor, 4-dentate lacinia mobilis, and eight raker setae on left mandible; with 5-dentate incisor and minutely dentate lacinia mobilis on right mandible, molar well developed, triturative; palp asymmetrical, composed of three articles, 3^rd^ article distally rounded, 0.8× as long as 2^nd^ article, with setae extending along most of posteriodistal margin.

Maxilla 1 (Fig. 4K, L) inner lobe small, subrectangular, produced laterodistally, with seven plumose setae; outer lobe nine dentate robust setae on apical margin; palp 2^nd^ article slightly swollen laterally, apex with six dentate robust setae.

Maxilla 2 (Fig. 4M) inner lobe with an oblique row of plumose setae on surface; outer lobe slightly larger than inner lobe.

**Figure 5:**
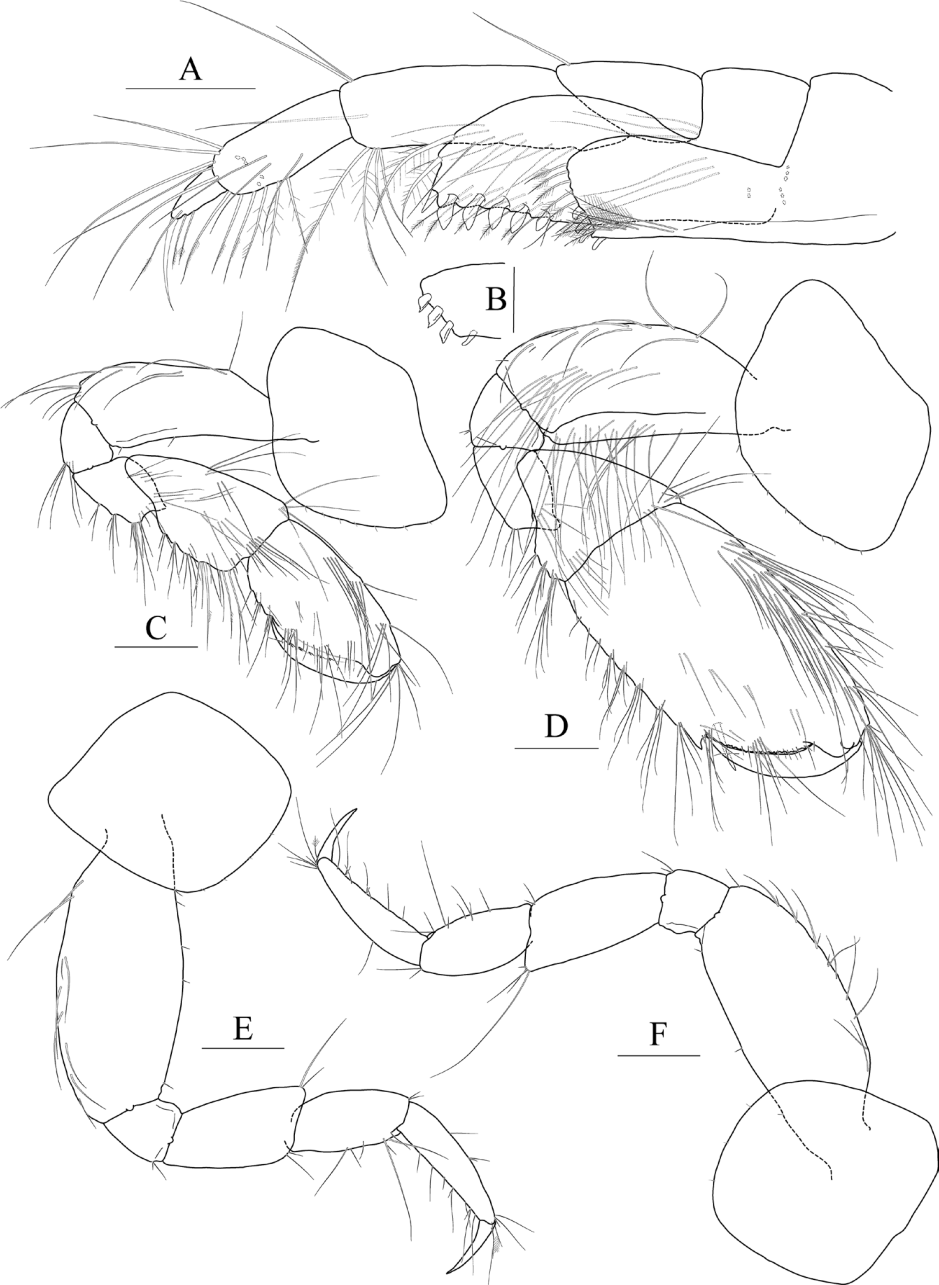
*Latigammaropsis careocavata* sp. nov., holotype, NIBRIV0000806529, male, 7.5 mm, Sogueulbi-do Island, South Korea. **A, B** maxilliped **C** gnathopod 1 **D** gnathopod 2 **E** pereopod 3 **F** pereopod 4. Scale bars: 0.05 mm (**B**), 0.1 mm (**A**), 0.2 mm (**C–F**).

Maxilliped (Fig. 5A, B) inner lobe subrectangular, with three dentate robust setae apically and one medial robust seta subdistally; outer lobe semiovoid, apex beyond half of 2^nd^ palp article, lined with eight robust setae on medial to apical margins; palp composed of four articles, 3^rd^ article a little expanded distally, half as long as 2^nd^ article, 4^th^ article 0.4× as long as 3^rd^ article, with elongate seta apically (1.3× as long as 4^th^ article).

Gnathopod 1 (Fig. 5C) setose with elongate setae; coxa subrhomboid, 0.9× as wide as long, weakly produced anterioventrally, rounded ventrally; basis subtrapezoidal, swollen posteriodistally; carpus subtrapezoidal, 0.9× as long as basis, half as wide as long, with carpal lobe blunt; propodus subovoid, as long and wide as carpus, palm oblique, slightly convex, minutely serrated, not defined distinctively, with one robust seta medially; dactylus elongate, 0.7× as long as propodus, inner margin minutely serrated, with five teeth.

Gnathopod 2 (Fig. 5D) stout, 1.3× as long gnathopod 1, densely setose with elongate setae; coxa subrhomboid, as long as wide; basis subtrapezoidal, half as wide as long, anterior margin with lateral and medial borders forming weak lobes distally, posterior margin swollen; carpus 0.7× as long as basis, with carpal lobe not free from propodus posterior margin; propodus 1.3× as long as basis, 0.6× as wide as long, anterior and posterior margins subparallel, palmar margin oblique, half as long as posterior margin, slightly convex, weakly serrated, without excavation, defined by one stout spine, with one elongate robust seta medially; dactylus fitting palm.

Pereopod 3 (Fig. 5E) rarely setose than gnathopods; coxa subquadrate, 0.9× as long as that of gnathopod 2; basis expanded, 0.7× as wide as coxa, posterior margin more swollen in the middle, along with elongate setae; merus 0.4× as long as basis, expanded anteriodistally, half as wide as long; carpus as long as merus, not expanded; propodus half as long as basis, diminished distally; dactylus falcate, 1.2× as long as propodus.

Pereopod 4 (Fig. 5F) similar to pereopod 3, except for longer merus (half as long as basis).

**Figure 6:**
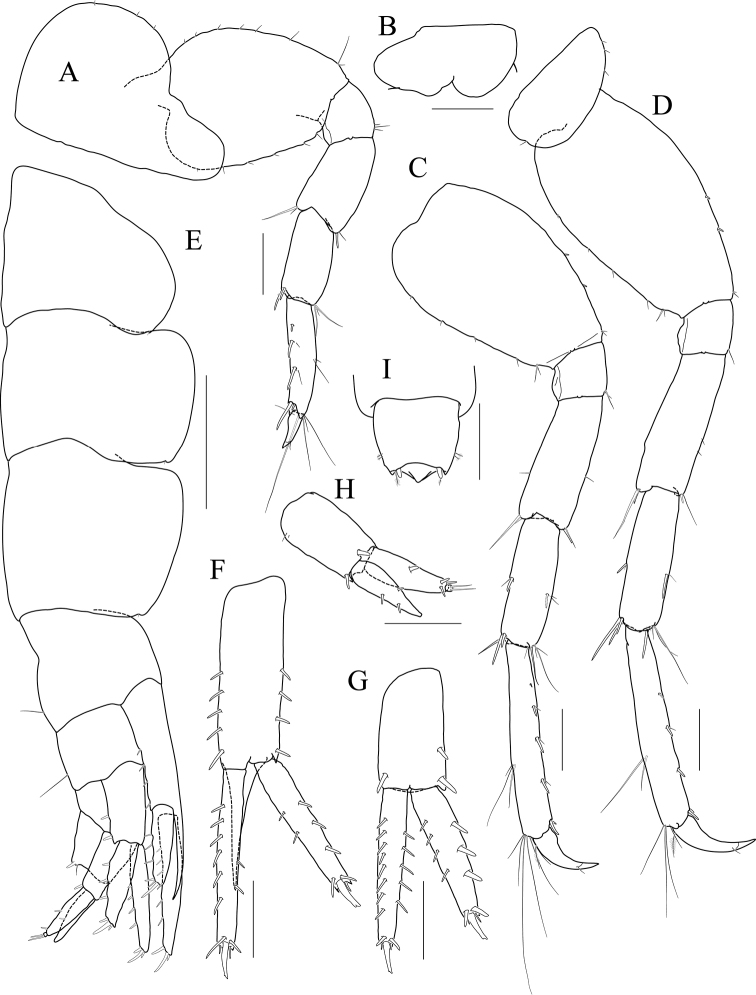
*Latigammaropsis careocavata* sp. nov., holotype, NIBRIV0000806529, male, 7.5 mm, Sogueulbi-do Island, South Korea. **A** pereopod 5 **B, C** pereopod 6 **D** pereopod 7 **E** pleonites and urosomites, lateral **F** uropod 1 **G** uropod 2 **H** uropod 3 **I** telson. Scale bars: 0.2 mm (**A–D, F–I**), 0.5 mm (**E**).

Pereopod 5 (Fig. 6A) coxa bilobed, anterior lobe larger than posterior lobe, expanded and rounded ventrally; basis as wide as coxa anterior lobe, 0.8× as wide as long, anterior margin convex, posterior margin extremely expanded proximally, merus 0.6× as long as basis, slightly expanded anteriodistally; carpus subrectangular, 0.9× as long as merus, half as wide as long; propodus 1.3× as long as carpus, with three robust setae on posterior margin, with one pair of distal locking setae extremely unequal in length; dactylus 0.4× as long as propodus.

Pereopod 6 (Fig. 6B, C) 1.3× as long as pereopod 5; coxa 0.7× as wide as that of pereopod 5; bilobed, anterior lobe expanded downwards, posterior lobe as large as anterior lobe, expanded posterioventrally; basis subovoid, anterior margin evenly rounded, posterior margin expanded proximally, 0.6× as wide as long; merus slightly expanded distally, 0.4× as wide as long, 0.6× as long as basis; carpus rectangular, 0.9× as long as merus, 0.3× as wide as long; propodus 1.5× as long as carpus, with a pair of distal locking setae unequal in length, with a group of five setae (longest seta 0.7× as long as propodus) at anteriodistal corner; dactylus falcate, 0.4× as long as propodus.

Pereopod 7 (Fig. 6D) similar and 1.2× as long as pereopod 6; coxa unilobed, as wide as that of pereopod 6; slightly expanded posterioventrally; basis subovoid, as wide and 1.1× as long as that of pereopod 6, anterior margin evenly rounded, posterior margin expanded proximally; merus slightly expanded distally, 0.3× as wide as long, 0.6× as long as basis; carpus rectangular, as long as merus, 0.3× as wide as long; propodus 1.4× as long as carpus, with a pair of distal locking setae unequal in length at anteriodistal corner; dactylus falcate, 0.4× as long as propodus.

Epimera 1–3 each with a small notch bearing minute seta at posterioventral corner. Epimeron 1 rounded ventrally. Epimera 2 and 3 subrectangular (Fig. 6E).

Uropod 1 (Fig. 6F) peduncle with a well-developed distoventral spine, 0.7× as long as peduncle, with four dorsomedial and four dorsolateral robust setae on distal half margin; outer ramus 0.8× as long as peduncle, with three dorsolateral and two dorsomedial robust setae, apex blunt bearing one group of robust setae; inner ramus 0.9× as long as peduncle, with two dorsolateral and six dorsomedial robust setae, apex blunt bearing one group of robust setae.

Uropod 2 (Fig. 6G) 0.7× as long as uropod 1; peduncle 0.6× as long as that of uropod 1, without distoventral spine, with one dorsomedial and two dorsolateral robust setae distally; outer ramus 1.1× as long as peduncle, with three dorsolateral and four dorsomedial robust setae, apex blunt bearing one group of robust setae; inner ramus 1.3× as long as peduncle, with five dorsolateral and eight dorsomedial robust setae, apex blunt bearing one group of robust setae.

Uropod 3 (Fig. 6H) 0.7× as long as uropod 2; peduncle 0.8× as long as that of uropod 2, with two robust setae distally; outer ramus as long as peduncle, biarticulated, 2^nd^ article vestigial, with two elongate setae subdistally; inner ramus 0.9× as long as peduncle, plump proximally but tapering distally.

Telson (Fig. 6I) subtrapezoidal in dorsal view, apex acute, with one robust seta on each side.

**Figure 7:**
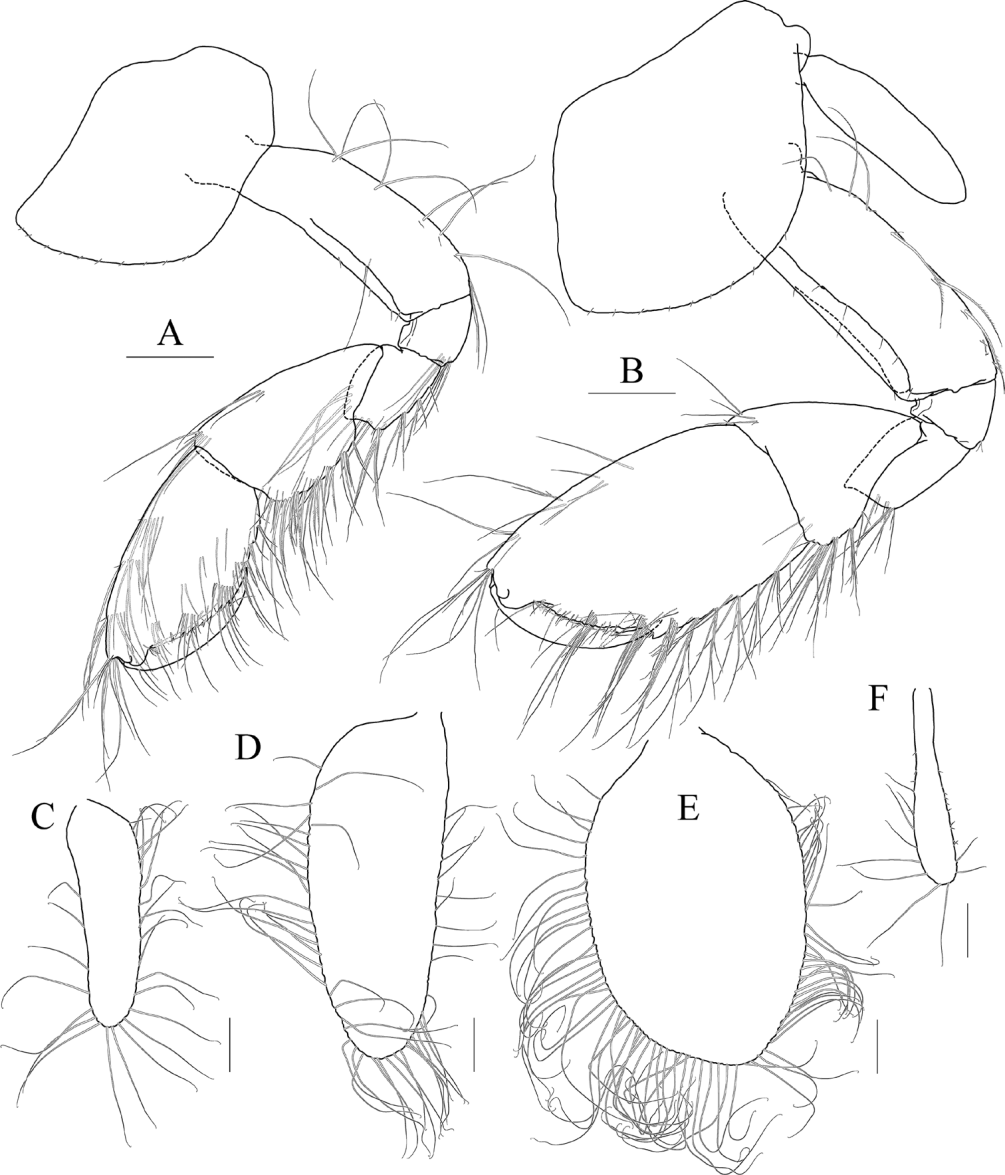
*Latigammaropsis careocavata* sp. nov., paratype, NIBRIV0000848929, female, 6.7 mm, Sogueulbi-do Island, South Korea. **A** gnathopod 1 **B** gnathopod 2 **C–F** oostegites of gnathopod 2–pereopod 5. Scale bars: 0.2 mm.

***Paratype female.*** Gnathopod 1 (Fig. 7A) not sexually dimorphic between both sexes.

Gnathopod 2 (Fig. 7B) similar to that of holotype male in size, less setose anteriorly; basis posterior margin less swollen; palm defining robust seta smaller than that of holotype male.

Oostegites (Fig. 7C–F) those of gnathopod 2 and pereopod 3 elongate; that of pereopod 4 ovoid, widest; that of pereopod 5 small, half as long as that of pereopod 4, linear.

####### Remarks.

The genus *Latigammaropsis* was established by Myers (2009) based on the ‘*atlantica*’ group of *Gammaropsis* sensu lato, and has been diagnosed by having a different shape of uropod 3: peduncle is shorter and broader; outer ramus is blunt-ended with a vestigial 2^nd^ article bearing two fine setae; and inner ramus is subequal to or shorter than outer ramus, narrowing distally and with a single small robust seta inserted at its tip (Barnard 1965, 1970; Myers 1995, 2009). The Korean material examined in this study also shows these diagnostic characters and could be readily assigned to the genus *Latigammaropsis*. Moreover, this material was identified as a new species, with specimens notably characterized by the absence of excavations on the palmar margin of gnathopod 2 in both sexes. Usually, mature males of the genus *Latigammaropsis* have an excavated gnathopod 2 palmar margin and a smooth margin has been considered a feature of immature specimens (Barnard 1970). Unfortunately, the authors of this study only described one holotype male and one paratype female, so it is unknown if intraspecific variations are related to maturity in both sexes. However, we certainly confirmed their maturity based on the presence of lageniform eyes in both sexes as a sign of adulthood (Barnard 1965), and the ovigerous paratype female having a similar shaped gnathopod 2.

The shape of gnathopod 2 between both sexes is quite similar to each other, except for the more setose basis and propodus in males. A lack of strong sexual dimorphism in gnathopod 2 was described in *Latigammaropsis gemina* (Myers, 1995) and *Latigammaropsis christenseni* (Myers, 1995). However, *Latigammaropsis careocavata* sp. nov. differs from these species by the absence of an excavated palmar margin and the strongly setose basis and propodus of gnathopod 2 in males (Myers 1995).

Until now, all known species of this genus have been recorded from tropical regions (Stebbing 1888; Schellenberg 1925, 1938; Barnard 1965, 1970; Ruffo 1969; Ledoyer 1978 ; Myers 1995, 2009, 2014; Myers and Nithyanandan 2016). This is the first record of the genus *Latigammaropsis* from Korean waters as well as from outside of the tropics.

##### Genus *Photis* Krøyer, 1842

###### Photis bronca sp. nov.

Taxon classificationAnimaliaAmphipodaPhotidae

93FECD02-FCD5-588C-ADAD-C77C85642F46

http://zoobank.org/CE27564C-91FB-47A7-8500-9740799302A0

**Figure 8:**
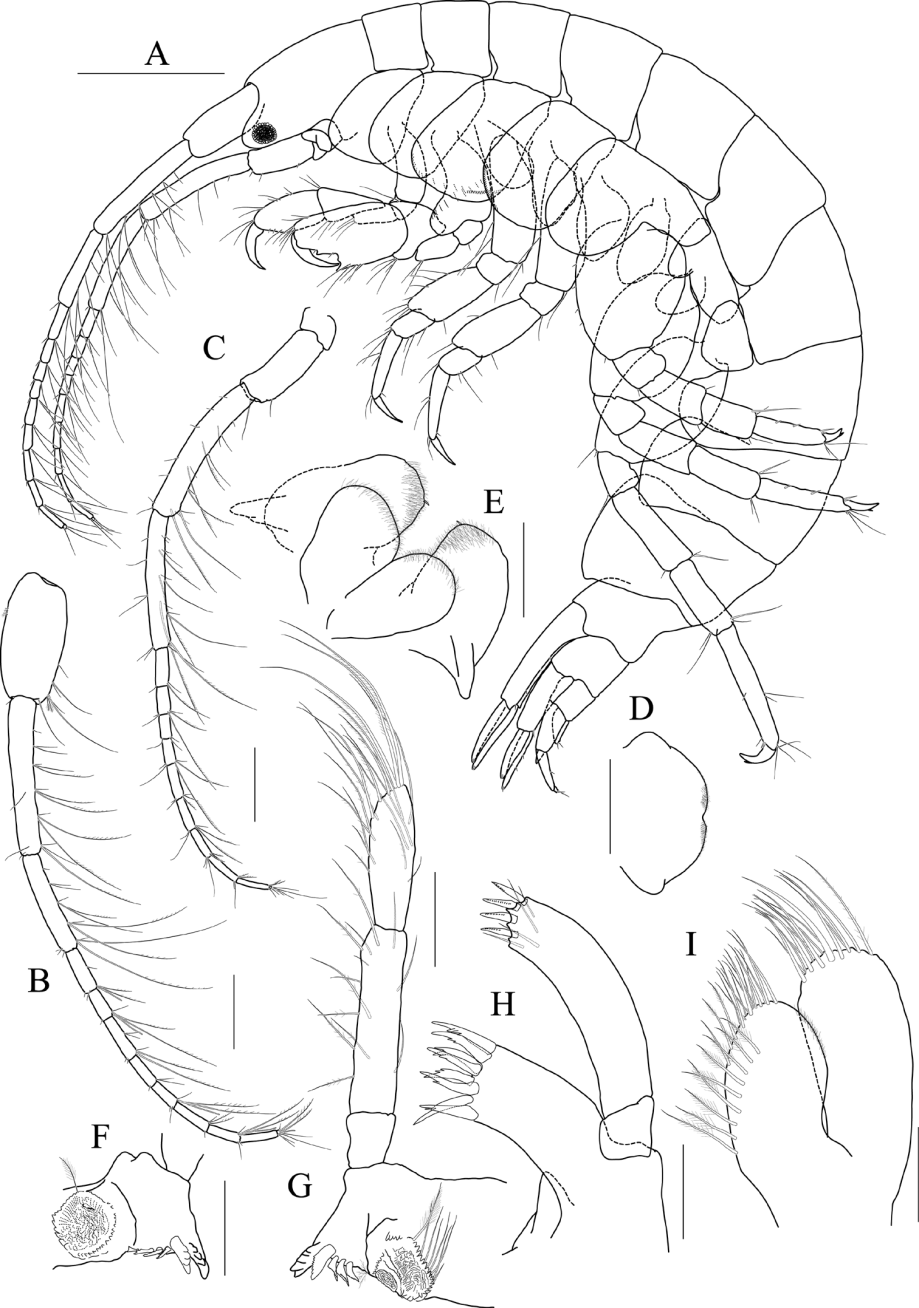
*Photis bronca* sp. nov., holotype, NIBRIV0000848472, male, 5.8 mm, Daryeo-do Island, South Korea. **A** habitus **B** antenna 1 **C** antenna 2 **D** upper lip **E** lower lip **F** left mandible **G** right mandible **H** maxilla 1 **I** maxilla 2. Scale bars: 0.1 mm (**D–I**), 0.2 mm (**B, C**), 0.5 mm (**A**).

**Figure 9:**
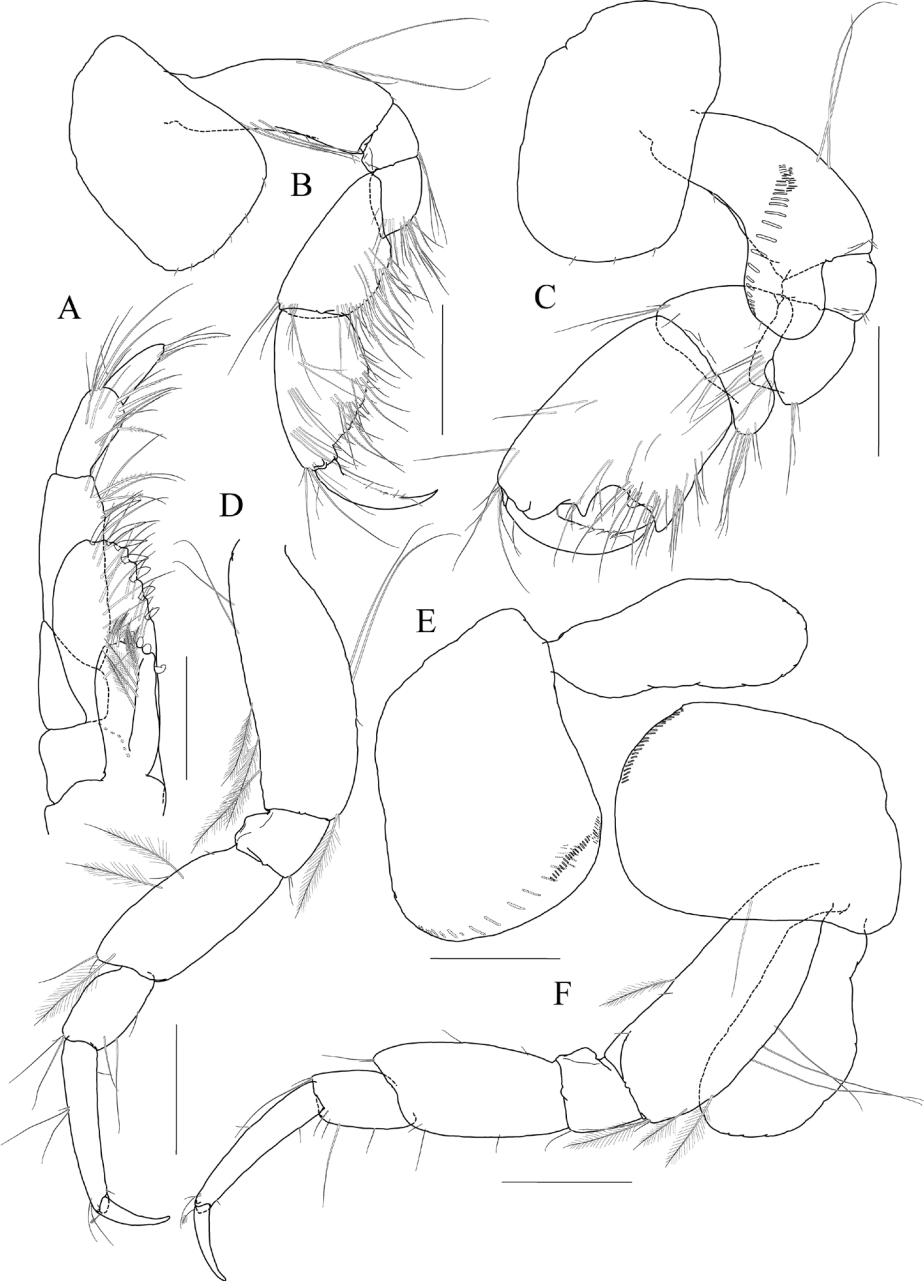
*Photis bronca* sp. nov., holotype, NIBRIV0000848472, male, 5.8 mm, Daryeo-do Island, South Korea. **A** maxilliped **B** gnathopod 1 **C** gnathopod 2 **D** pereopod 3 **E** coxa of pereopod 3 **F** pereopod 4. Scale bars: 0.1 mm (**A**), 0.2 mm (**B–F**).

**Figure 10:**
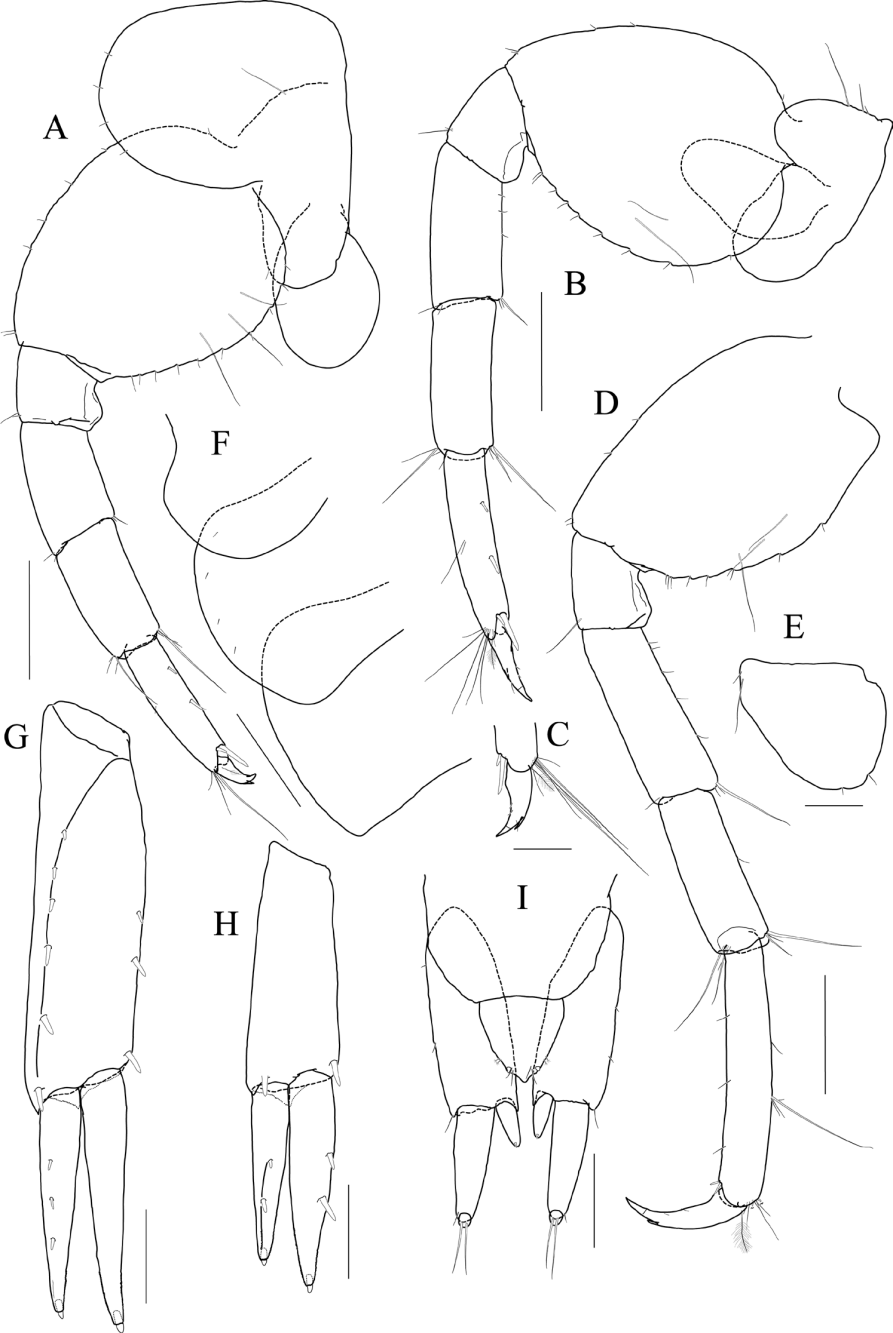
*Photis bronca* sp. nov., holotype, NIBRIV0000848472, male, 5.8 mm, Daryeo-do Island, South Korea. **A** pereopod 5 **B**, pereopod 6 **C** dactylus of pereopod 6 **D** coxa of pereopod 7 **E** pereopod 7 **F** uropod 1 **G** uropod 2 **H** uropod 3 and telson. Scale bars: 0.1 mm (**C, E, G–I**), 0.2 mm (**A, B, D, F**).

**Figure 11:**
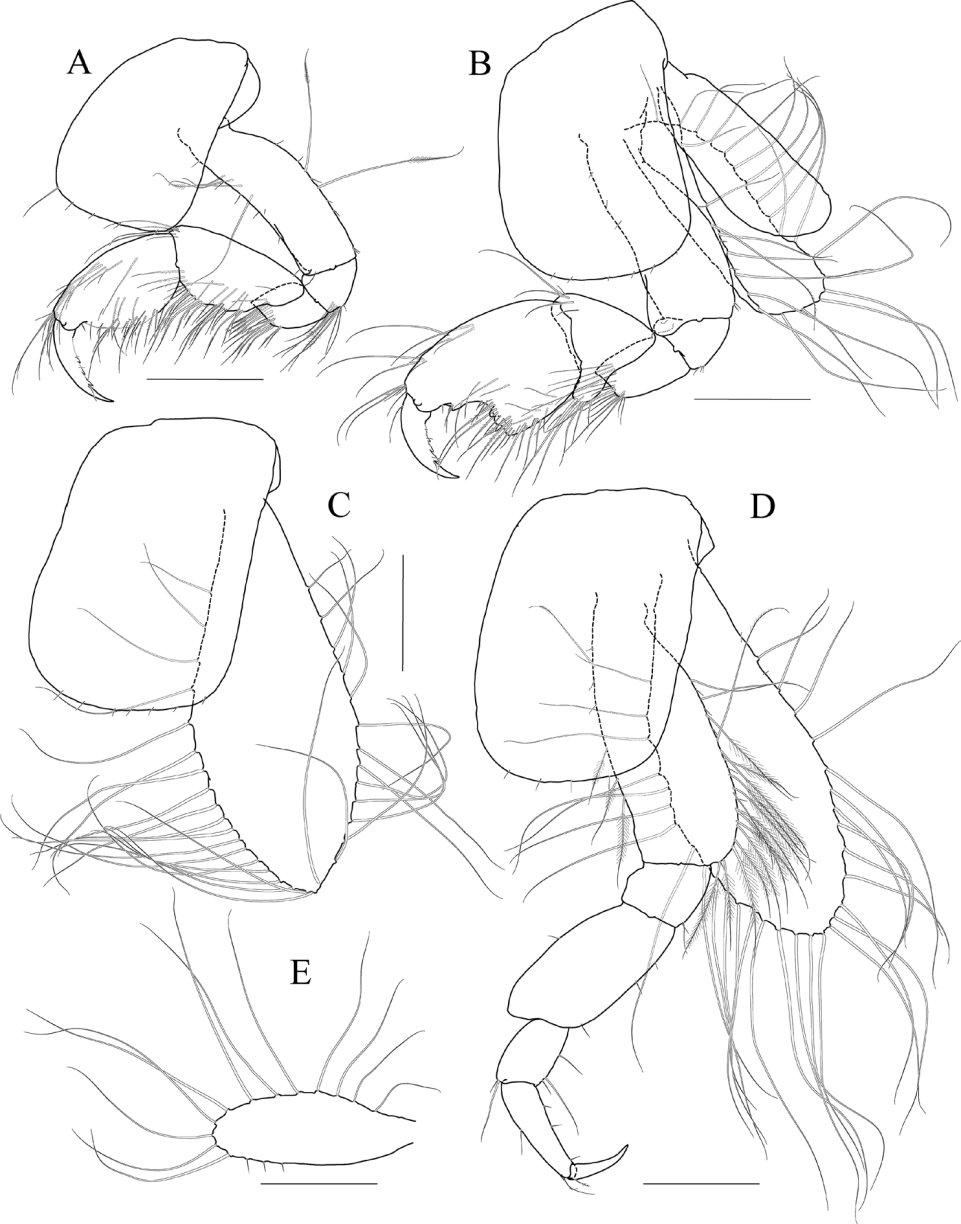
*Photis bronca* sp. nov., paratype, NIBRIV0000848473, female, 5.3 mm, Daryeo-do Island, South Korea. **A** gnathopod 1 **B** gnathopod 2 **C** coxa and oostegite of pereopod 3 **D** pereopod 4 **E** oostegite of pereopod 5. Scale bars: 0.2 mm.

Figs 8 , 9 , 10 , 11

Photis longicaudata : Nagata 1965: 310, fig. 35; Hirayama 1984: 42, fig. 71; Kim et al. 2005: 3, figs 1, 2; Ren 1992: 259, fig. 28; 2000: 141, fig. 4; 2006: 398, fig. 171 [non P. longicaudata (Spence Bate & Westwood, 1862)].

####### Etymology.

The epithet of the specific name, *bronca*, has its origin from the Latin word *broncus*. This name refers to the bearing of a quadrate tooth medially on the gnathopod 2 palmar margin in both sexes.

####### Material examined.

Holotype: ♂ (5.8 mm), NIBRIV0000848472; Daryeo-do Island, Bukchon, Jeju-do, South Korea (34°33'27"N, 126°41'49"E); 30 Nov, 2012; grab sampler (about 20 m depth), by Prof. HY Soh. Paratypes: 2 ♂♂ (2.3 and 2.5 mm), 3 ♀♀ (ovigerous; 3.2–5.3 mm), NIBRIV0000848473; same data as holotype.

Additional materials: 3 ♂♂ 2 ♀♀, NIBRIV0000848930, Saekkiseom, Ae-do Island, Mijo-myeon, Gyeongsangnam-do, South Korea (34°41'31"N, 128°02'00"E), 1 May 2009, Scuba diving (about 20 m depth), by TW Jung; 2 ♂♂, NIBRIV0000848931, Jeolmyeongyeo, Chuja-do Island, Jeju-do, South Korea (33°52'04"N, 126°18'43"E), 12 Jul, 2016, Scuba diving (about 16 m depth), by CH Yi; 3 ♂♂ 2 ♀♀, NIBRIV0000848932, Yenae-ri, Goheung-gun, Jeollanam-do, South Korea (34°27'24"N, 127°31'16"E), 28 Mar 2017, washing macro-algae, by SH Kim.

####### Diagnosis.

In both sexes, gnathopod 2 propodus palmar margin with subrectangular cavity bearing one rectangular tooth medially. Male gnathopod 2 basis lateral margin forming a well-developed sac-like lobe. Stridulated ridges only present on male gnathopod 2 basis and coxae 3 and 4 (absent in females).

####### Description.

**Holotype male.** Head (Fig. 8A) as long as pereonites 1 and 2 combined; lateral cephalic lobe rounded; eye circular, moderate in size, located in the middle of lateral lobe; antennal sinus deep.

Antenna 1 (Fig. 8B) 0.4× as long as body; peduncle 1^st^ article stout, 0.7× as long as head; 2^nd^ article slender, 1.3× as long as 1^st^ article; 3^rd^ article 0.6× as long as 2^nd^ article; accessory flagellum absent; flagellum 0.8× as long as peduncle 1^st^–3^rd^ articles combined, composed of ten articles (terminal article rudimentary).

Antenna 2 (Fig. 8C) 1.1× as long as antenna 1; peduncle 3^th^ article reaching end of lateral cephalic lobe; 4^th^ article as long as 2^nd^ article of antenna 1; 5^th^ article as long as 4^th^ article; flagellum 0.7× as long as peduncle 3^rd^–5^th^ articles combined, composed of ten articles (terminal article rudimentary).

Upper lip (Fig. 8D) convex anteriorly, with notch in the middle, covered with minute setae.

Lower lip (Fig. 8E) inner lobe subovoid, outer lobe apex rounded, covered with minute setae, with one robust seta at mediodistal corner; mandibular process well developed.

Mandibles (Fig. 8F, G) with 4-dentate incisor, 4-dentate lacinia mobilis, and four raker setae on left mandible; with 5-dentate incisor, minutely dentate lacinia mobilis, and three raker setae on right mandible; molar well developed, triturative, with seven setae along the distal margin of right mandible; palp asymmetrical, composed of three articles, 3^rd^ article distally rounded, 0.8× as long as 2^nd^ article, with setae extending along most of posteriodistal margin.

Maxilla 1 (Fig. 8H) inner lobe small, without minute setae; outer lobe with eight dentate robust setae on apical margin; palp biarticulated, distal article curved, with four dentate setae on apical margin.

Maxilla 2 (Fig. 8I) inner lobe with an oblique row of plumose setae on surface; outer lobe longer and slightly dilated distally than inner lobe.

Maxilliped (Fig. 9A) inner lobe subrectangular, a little expanded distally, with three nodular setae apically and one medial nodular seta subdistally; outer lobe exceeding half of palp 2^nd^ article, lined with seven robust setae along apex to medial margin; palp composed of four articles, 3^rd^ article slightly expanded distally, half as long as 2^nd^ article, 4^th^ article 0.8× as long as 3^rd^ article, with elongate seta apically (as long as 4^th^ article).

Gnathopod 1 (Fig. 9B) coxa subtrapezoidal, slightly extended distally, 0.7× as wide as long; basis as long as coxa, anterior margin lateral border forming weak lobe distally, with three elongate setae subproximally, posterior margin with three elongate setae at the middle; carpus subtrapezoidal, 0.7× as long as basis, half as wide as long, posterior lobe blunt; propodus as long and wide as carpus, rounded anteriorly, with minute serrations irregularly along palm and posterior margin, palm 0.6× as long as posterior margin, defined by one robust seta medially; dactylus 0.7× as long as propodus, exceeding palm, inner margin serrated, with two teeth.

Gnathopod 2 (Fig. 9C) stout, coxa subrectangular, 0.6× as wide as long, slightly extended and rounded anterioventrally; basis anterior margin lateral borders forming well-developed lobe distally (sac-like lobe reaching middle of merus) bearing oblique stridulated ridges on surface; ischium with small anterior lobe only; merus subrectangular, 0.4× as long as basis; carpus stout, anterior margin convex, carpal lobe well developed; propodus stout, as long as basis, 0.7× as wide as long, slightly widened distally, palmar margin defined by one large blunt spine, with small obtuse spine near dactylus base and robust seta near defining spine, concave subrectangularly, bearing quadrate tooth medially; dactylus half as long as propodus, with two teeth on inner margin.

Pereopod 3 (Fig. 9D, E) coxa widened distally, produced anterioventrally, 0.8× as wide as long, with stridulated ridges on medial surface and short stridulated ridges near the posterioventral corner on lateral surface submarginally; basis 0.4× as wide as long, with three plumose setae along distal half of anterior margin, posterior margin expanded, evenly rounded, with two elongate setae at the middle, with a pair of plumose and minute setae at distal corner; merus 0.7× as long as basis, anterior margin weakly expanded, with two plumose setae submarginally, distal corner somewhat produced, with one plumose seta subapically; carpus subrectangular, half as long as and 0.7× as wide as merus; propodus slender, diminished distally, 0.6× as long as basis; dactylus 0.4× as long as propodus.

Pereopod 4 (Fig. 9F) coxa slightly widened distally, as long as that of pereopod 3, with stridulated ridges along anterioventral corner more oblique than that of female; basis 0.4× as wide as long, with one plumose seta at the middle of anterior margin, posterior margin expanded, evenly rounded, with three elongate setae at the middle, with three plumose setae distally; merus 0.7× as long as basis, anterior margin weakly expanded, distal corner somewhat produced; carpus half as long as and 0.7× as wide as merus, propodus slender, diminished distally, 0.6× as long as basis; dactylus 0.4× as long as propodus.

Pereopod 5 (Fig. 10A) coxa bilobed, large, anterior lobe subovoid, 0.7× as wide, expanded ventrally, posterior lobe small, expanded backwards; basis subovoid, broad, more expanded proximally, 0.8× as wide as long; merus subrectangular, half as long as basis, half as wide as long; carpus subrectangular, as long and wide as merus; propodus 1.1× as long as carpus, with a pair of distal locking setae unequal in length (longer seta 0.8× as long as dactylus), with a group of four setae (longest seta 0.6× as long as propodus) at anteriodistal corner; dactylus 0.4× as long as propodus, armed with one accessory cusp on outer margin.

Pereopod 6 (Fig. 10B, C) 1.2× as long as pereopod 5; coxa bilobed, anterior lobe small, posterior lobe weakly dilated posterioventrally; basis subovoid, 0.8× as wide as long, anterior margin convex, posterior margin slightly dilated proximally; merus half as long as basis, 0.4× as wide as long; carpus as long and 0.9× as wide as merus; propodus 1.3× as long as carpus, with one pair of distal locking setae unequal in length (longer seta 0.6× as long as dactylus), with one group of five setae (longest seta half as long as propodus) at anteriodistal corner; dactylus 0.4× as long as propodus, armed with one accessory cusp on outer margin.

Pereopod 7 (Fig. 10D, E) 1.2× as long as pereopod 6; coxa unilobed, dilated posterioventrally; basis subovoid, 0.8× as wide as that of pereopod 6, 0.7× as wide as long, anterior margin rather convex, posterior margin with one blunt extension proximally; merus subrectangular, a little extended posteriodistally, 0.6× as long as basis; carpus rectangular, 0.9× as long as merus, 0.4× as long as wide; propodus 1.6× as long as carpus, with one pair of distal locking setae subequal (smaller than those of pereopods 5 and 6); dactylus 0.4× as long as propodus, armed with one accessory cusp on outer margin.

Epimeron 1 slightly extended anterioventrally. Epimera 2 and 3 with each posterioventral corner produced backwards, but not acute (Fig. 10F).

Uropod 1 (Fig. 10G) peduncle without distoventral spine, but bearing small acute extension, with six dorsolateral and three dorsomedial robust setae; inner ramus 0.6× as long as peduncle, with one subapical seta only; outer ramus 0.9× as long as inner ramus, with three dorsolateral setae and one subapical seta.

Uropod 2 (Fig. 10H) 0.7× as long as uropod 1; peduncle 0.6× as long as that of uropod 1, with small acute extension distoventrally; inner ramus 0.9× as long as peduncle, with two dorsomedial setae and one subapical seta; outer ramus 0.8× as long as inner ramus, with two dorsolateral setae and one subapical seta.

Uropod 3 (Fig. 10I) 0.7× as long as uropod 2; peduncle 0.8× as long as that of uropod 2; outer ramus biarticular, 0.6× as long as peduncle, last article vestigial, with two elongate setae subapically; inner ramus scale-like, 0.3× as long as outer ramus.

Telson (Fig. 10I) subtriangular in dorsal view, with a pair of simple setae, a pair of sensory setae, and one nodular robust seta on each side.

***Paratype female.*** Gnathopod 1 (Fig. 11A) not sexually dimorphic.

Gnathopod 2 (Fig. 11B) stouter than female gnathopod 1, slightly smaller than that of male; coxa subrectangular, 1.5× as long as wide, evenly rounded anterioventrally, oostegite 1.2× as long as basis, 2.9× as long as wide; basis anterior margin lateral borders forming lobe distally, but smaller than that of male (not exceeding ischium), without stridulated ridges on surface; ischium with small anterior lobe only; merus rectangular, 0.4× as long as basis; carpus stout, anterior margin convex, carpal lobe well developed; propodus stout, 0.8× as long as basis, 0.7× as wide as long, not widened distally, palmar margin cavity subquadrate, bearing one quadrate tooth medially, proximal and defining spines weaker than those of male, with one robust defining seta medially; dactylus half as long as propodus, inner margin minutely serrated, with three teeth.

Pereopod 3 (Fig. 11C) not sexually dimorphic except for the absence of both medial and lateral stridulated ridges on coxa; oostegite 1.5× as long as that of gnathopod 2.

Pereopod 4 (Fig. 11D) coxa without stridulated ridges at anterioventral corner, basis posterior margin more expanded distally, with twelve plumose setae along distal half.

Pereopod 5 oostegite (Fig. 11E) half as long as that of pereopod 4.

####### Remarks.

**Figure 12:**
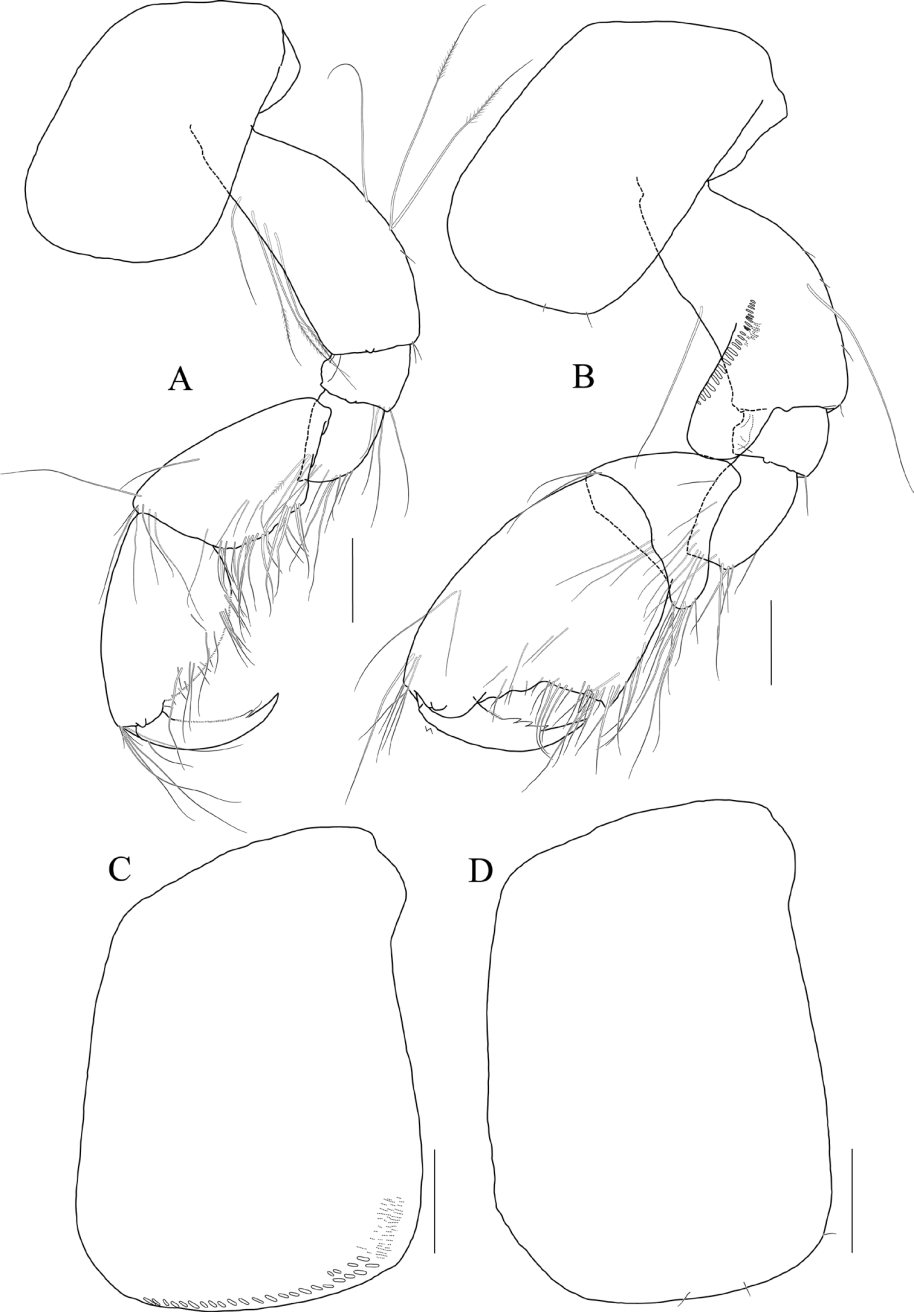
*Photis longicaudata* (Spence Bate & Westwood, 1862), lectotype, NHMUK 1911.20899, male. 3.2 mm, Shetland Islands, UK. **A** gnathopod 1 **B** gnathopod 2 **C** coxa of pereopod 3 **D** coxa of pereopod 4. Scale bars: 0.1 mm.

**Figure 13:**
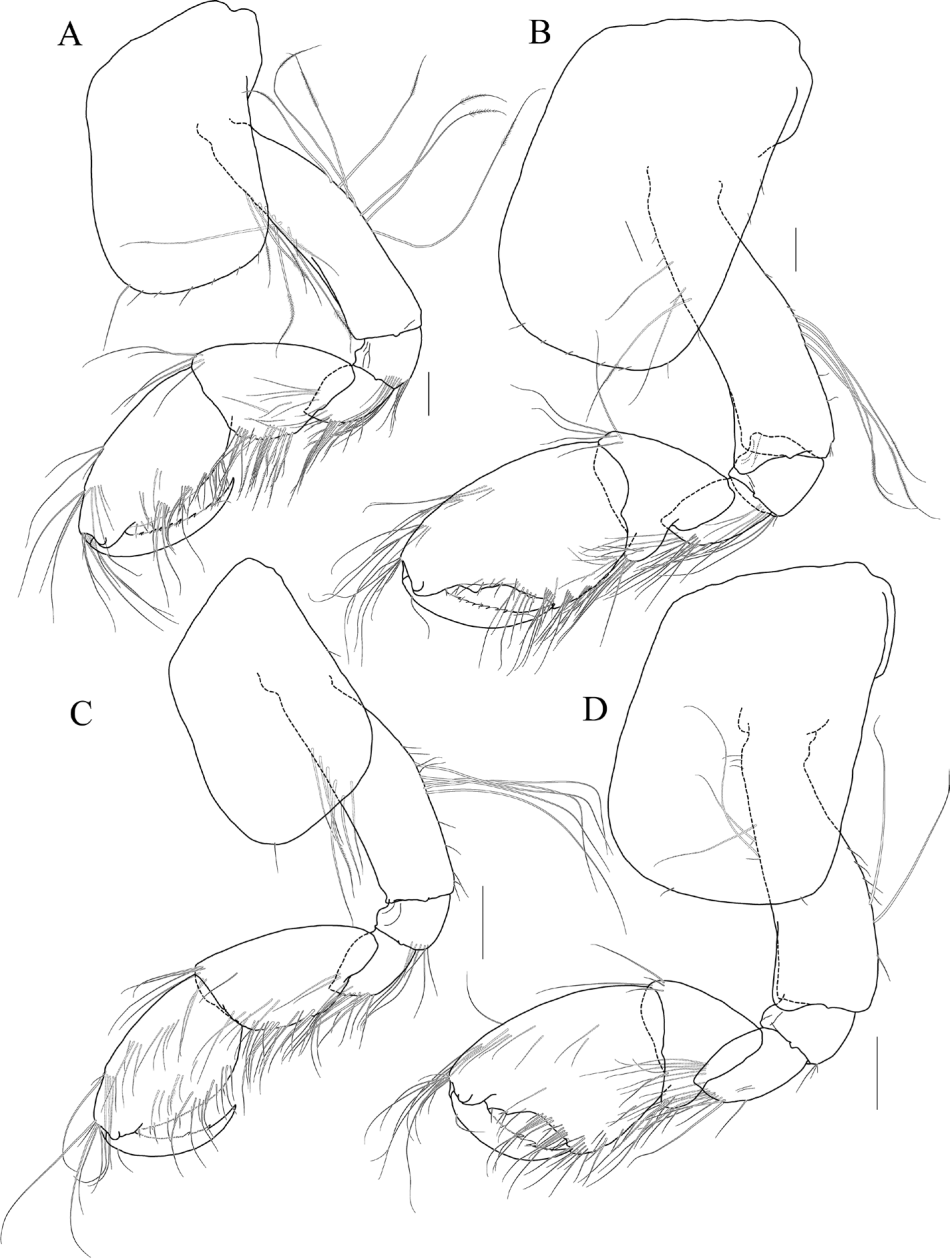
*Photis longicaudata* (Spence Bate & Westwood, 1862), paralectotype, NHMUK 1911.20890–20896, females. 5.3 mm (**A, B**) and 3.4 mm (**C, D**), Shetland Islands, UK **A** gnathopod 1 **B** gnathopod 2 **C** gnathopod 1 **D** gnathopod 2. Scale bars: 0.1 mm.

*Photis bronca* sp. nov. has been misidentified as *Photis longicaudata* (Spence Bate & Westwood, 1862) in the Far East including Korea (Kim et al. 2005), Japan (Nagata 1965; Hirayama 1984), and China (Ren 1992, 2000, 2006). The authors re-examined the syntype series of *P. longicaudata* deposited at the Natural History Museum, London (NHMUK 1911.20899–20906; Figs 12, 13) and several specimens, which were collected from the North Sea, at the Museum für Naturkunde Berlin (ZMB 11656) to confirm the validity of *P. longicaudata* in Korean waters. As a result, the material formerly identified as *P. longicaudata* from Japan and China, as well as the Korean material, is herein described as a new species based on the following differences when compared with the newly designated *P. longicaudata* lectotype (NHMUK 1911.20899; Fig. 12): gnathopod 1 palmar margin has a robust defining seta medially in both sexes (absent in the *P. longicaudata* lectotype); gnathopod 2 palmar margin has a quadrate tooth medially in both sexes (obviously absent in the *P. longicaudata* lectotype); the sac-like lobe of male gnathopod 2 basis is much larger than that of *P. longicaudata* (slightly exceeding the ischium in the *P. longicaudata* lectotype); the anterior margin of male gnathopod 2 propodus is a little rounded (slightly shorter and more swollen in the *P. longicaudata* lectotype); the palmar margin of male gnathopod 2 propodus has more produced spines (slightly produced in the *P. longicaudata* lectotype); male pereopod 3 coxa has a different pattern of stridulated ridges; the male pereopod 4 coxa has stridulated ridges on the anterior half of the ventral margin (absent in the *P. longicaudata* lectotype); and female gnathopod 2 palmar margin is concave subrectangularly (more oblique and recessed in the *P. longicaudata* lectotype) (Bate and Westwood 1863; Sars 1895; Nagata 1965; Hirayama 1984; Ren 1992, 2000, 2006; Kim et al. 2005; see Figs 12, 13).

It is well known that both *Photis aina* JL Barnard, 1970 and *Photis kapapa* JL Barnard, 1970 from the Hawaiian Islands have affinities with *P. longicaudata* in that gnathopod 2 has very well-developed (sac-like) anterior lobe of the basis (JL Barnard 1970). Among them, *Photis bronca* sp. nov. differs from *P. aina*, regarding the eyes being in the middle of the cephalic lobe (occupying the end of the cephalic lobe in *P. aina*), lacking the defining seta on the palmar margin of male gnathopod 1 (with one robust seta medially in *P. aina*), lacking posterioventral notches of epimera 2 and 3, and a shorter inner ramus of uropod 2 (about half length in *P. aina*) (Barnard 1970; Myers 1985, 2009). *Photis bronca* sp. nov. can be distinguished from *P. kapapa* by the following differences: the eyes occupying at the middle of the cephalic lobe (at the end of the cephalic lobe in *P. kapapa*), lacking stridulated ridges in both posterior margins of the coxae of gnathopods 1 and 2, 0.7 times as wide as long male gnathopod 2 (0.8 times in *P. kapapa*), the female palmar margin of gnathopod 2 with a small obtuse spine near the dactylus base (without spine in *P. kapapa*), the presence of one accessory cusp on pereopod 5 dactylus (two accessory cusps in *P. kapapa*), subacutely produced posterior margin of epimeron 3 (rounded in *P. kapapa*), and uropod 3 outer ramus with the length of 0.6 times as long as the peduncle (0.9 times in *P. kapapa*) (Barnard 1970; Ren 2006).

*Photis bronca* sp. nov. resembles *Photis davei* Myers, 2009 in the shapes of elongate propodus of pereopods 3 and 4 as well as the outlines of gnathopods in both sexes, but the former differs from the latter in the presence of a well-developed sac-like lateral lobe on the basis of male gnathopod 2 (present but very weak in *P. davei*) and not-elongate rami of uropods 1 and 2 (Myers 2009).

*Photis fischmanni* Gurjanova, 1951 also has a similar gnathopod 2 shape in both sexes, but *P. bronca* sp. nov. is clearly different from this species by elongate antenna 1 and 2 (0.4 times as long as the body in *P. bronca* sp. nov., but less than 0.3 times in *P. fischmanni*), less expanded carpus and propodus of gnathopod 1, and less setose appendages than *P. fischmanni* (Gurjanova 1951, 1955; Conlan 1983).

This new species differs from *Photis paeowai* Myers, 1995 and *Photis pirloti* Myers, 1985 by bearing a well-developed sac-like lateral lobe on the basis of male gnathopod 2 (present but rather weak in *P. paeowai* and *P. pirloti*) and a quadrate medial tooth on the palmar margin of gnathopod 2 in both sexes (absent in *P. paeowai* and *P. pirloti*) (Myers 1985, 1995).

###### Photis longicaudata

Taxon classificationAnimaliaAmphipodaPhotidae

(Spence Bate & Westwood, 1862)

1C503E05-12BA-548F-B6C3-012255687F6C

Figs 12 , 13

Eiscladus longicaudatus Spence Bate & Westwood, 1862: 412.
Heiscladus longicaudatus Norman, 1869: 284.
Heiscladius longicaudatus Norman, 1874: 269.
Photis longicaudata : Sars 1894: 571, pl. 203, fig. 1; Stebbing 1906: 608; Chevreux and Fage 1925: 310: Schellenberg 1942: 201; Lincoln 1979: 518, figs 237a, 248a–g; Myers and McGrath 1981: 766.

####### Material examined.

Lectotype: ♂, NHMUK 1911.20899. Shetlands, UK, 1861, by AM Norman. Paralectotypes: 7 specimens, NHMUK 1911.20900–20906. Same data as lectotype.

Additional material: 3 ♀♀, ZMB 11656. Nordsee, the North Sea, 1903, by Breidow.

####### Remarks.

The materials identified as *Photis longicaudata* from the Far East including Korea, Japan, and China were re-examined in the present study, and turned to the new species, *Photis bronca* sp. nov. See the remarks of *Photis bronca* sp. nov.

###### Photis longicarpus sp. nov.

Taxon classificationAnimaliaAmphipodaPhotidae

C85805AF-282A-5E6A-99F0-DBED3C28535D

http://zoobank.org/EF85D444-15FD-435E-8813-95EE183B8E4D

**Figure 14:**
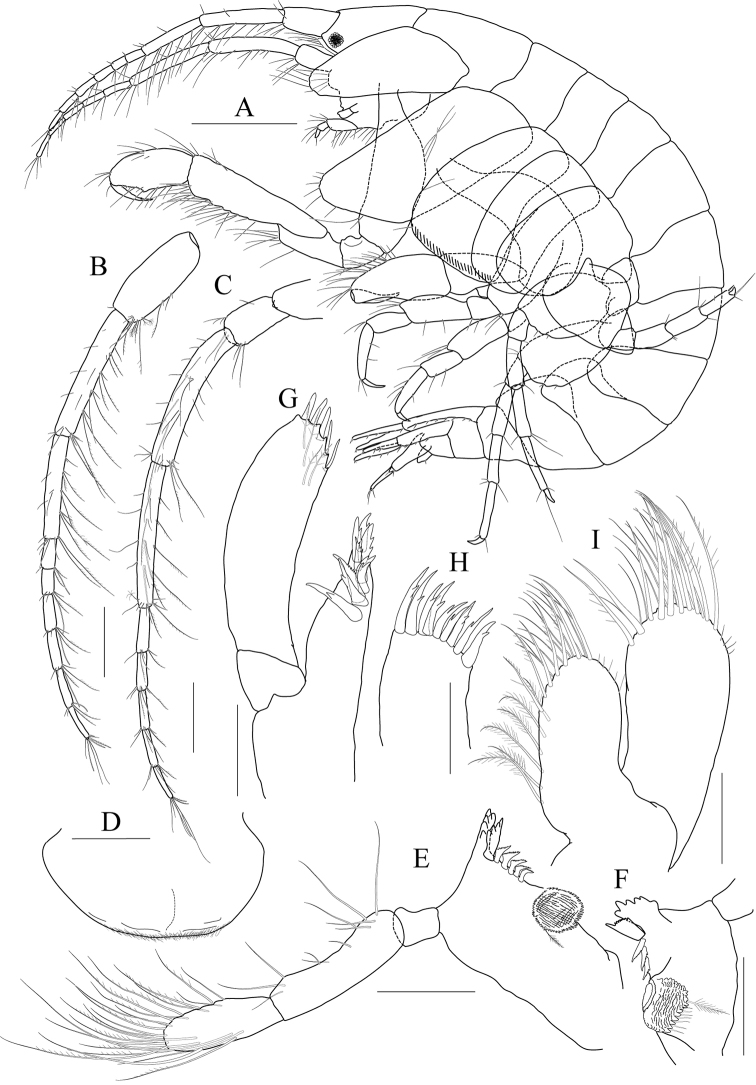
*Photis longicarpus* sp. nov., holotype, NIBRIV0000753910, male, 4.9 mm, Geomeunyeo, Jeju-do, South Korea. **A** habitus **B** antenna 1 **C** antenna 2 **D** upper lip **E** left mandible **F** right mandible **G, H** maxilla 1 **I** maxilla 2. Scale bars: 0.05 mm (**D, G–I**), 0.1 mm (**E, F**), 0.2 mm (**B, C**), 0.5 mm (**A**).

**Figure 15:**
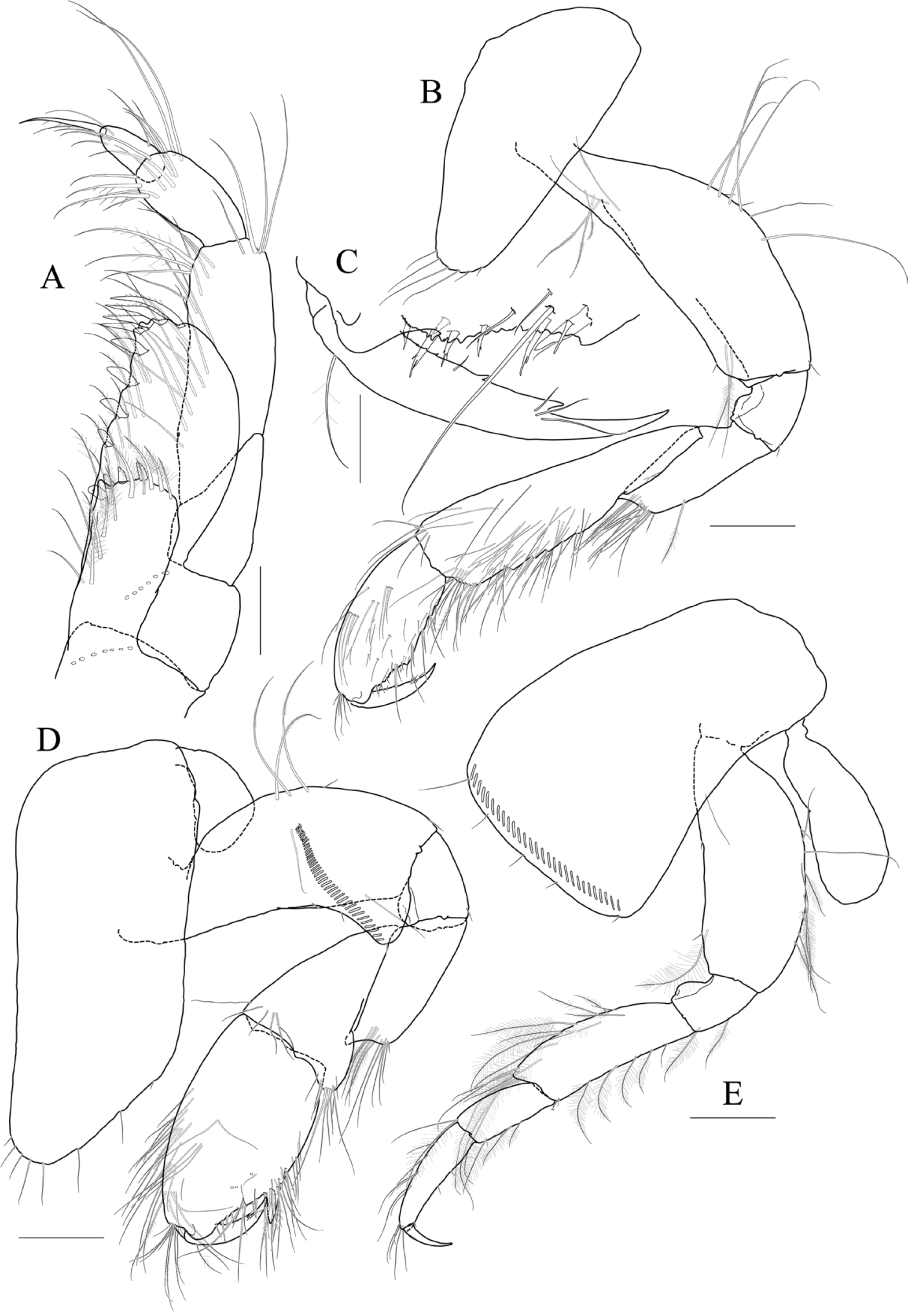
*Photis longicarpus* sp. nov., holotype, NIBRIV0000753910, male, 4.9 mm, Geomeunyeo, Jeju-do, South Korea. **A** maxilliped **B, C** gnathopod 1 **D** gnathopod 2 **E** pereopod 3. Scale bars: 0.05 mm (**A, C**), 0.2 mm (**B, D, E**).

**Figure 16:**
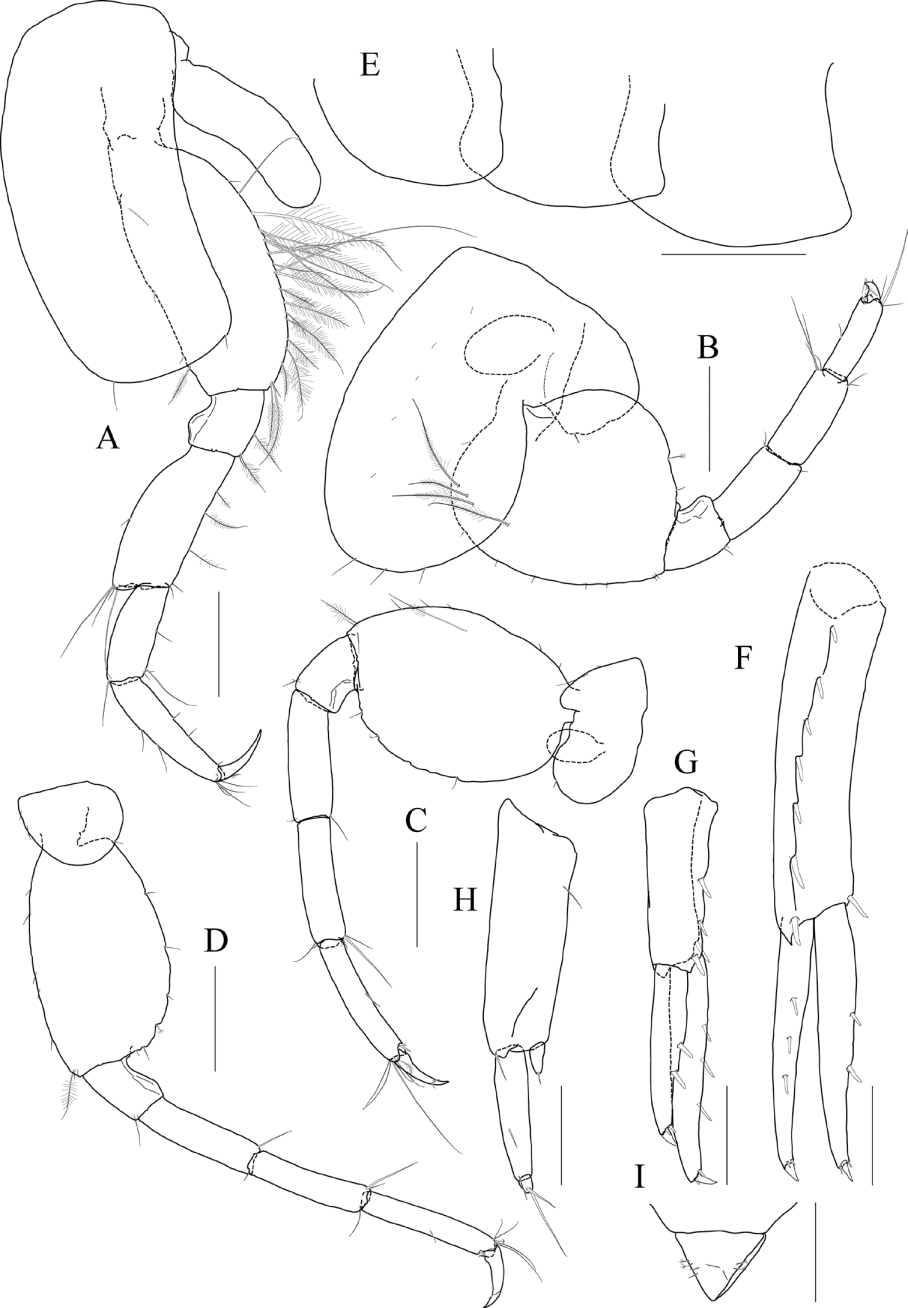
*Photis longicarpus* sp. nov., holotype, NIBRIV0000753910, male, 4.9 mm, Geomeunyeo, Jeju-do, South Korea. **A** pereopod 4 **B** pereopod 5 **C** pereopod 6 **D** pereopod 7 **E** pleonal epimera **F** uropod 1 **G** uropod 2 **H** uropod 3 **I** telson. Scale bars: 0.1 mm (**F–I**), 0.2 mm (**A–E**).

**Figure 17:**
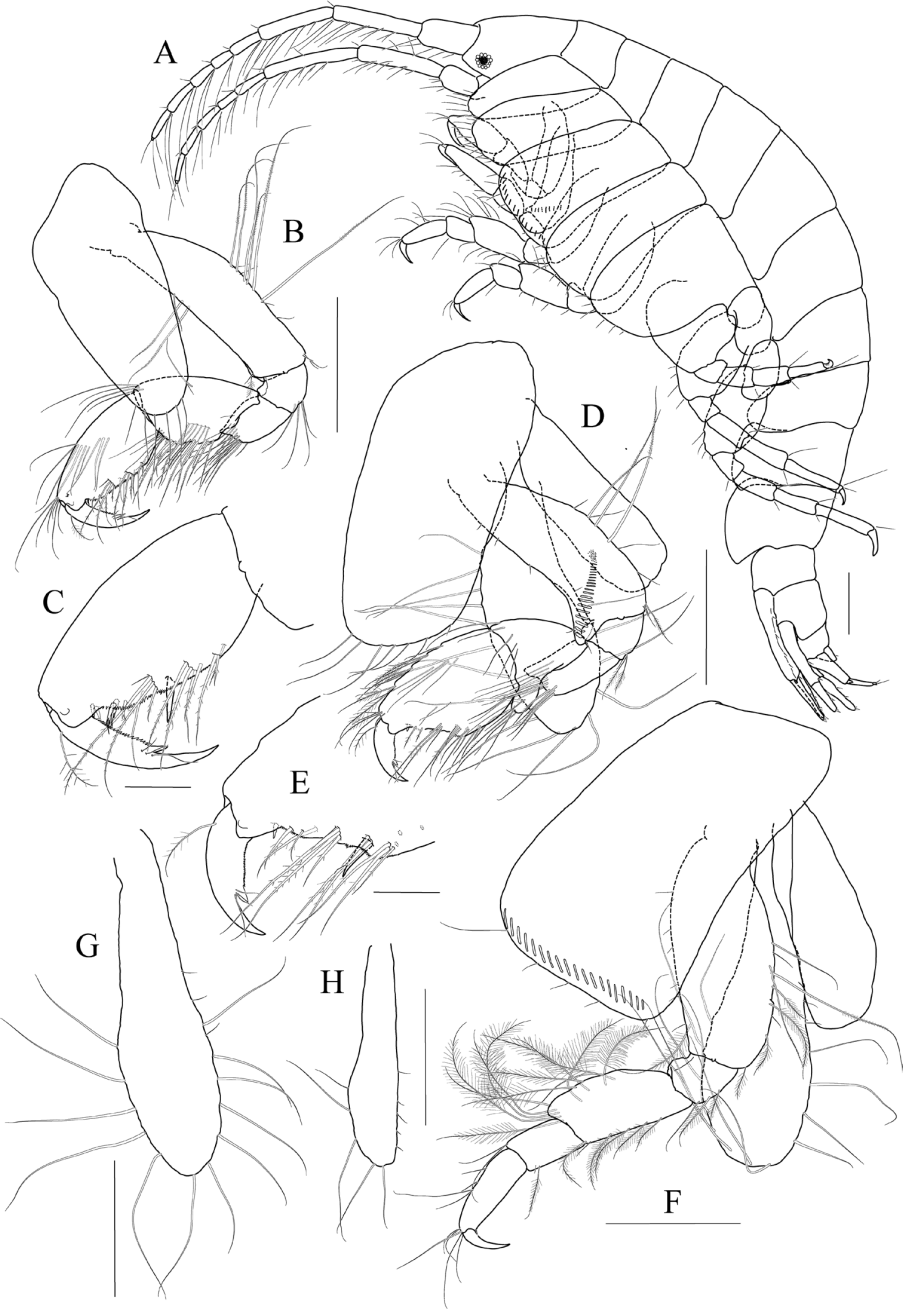
*Photis longicarpus* sp. nov., paratype, NIBRIV0000848928, female, 3.1mm, Geomeunyeo, Jeju-do, South Korea. **A** habitus **B, C** gnathopod 1 **D, E** gnathopod 2 **F** pereopod 3 **G, H** oostegites of pereopods 4 and 5. Scale bars: 0.05 mm (**C, E**), 0.2 mm (**A, B, D, F–H**).

Figs 14 , 15 , 16 , 17

####### Etymology.

The composite epithet of the specific name, *longicarpus*, is a combination of the Latin words *longus* and *carpus*, meaning elongate carpus. This name refers to the elongate shape of the carpus of gnathopod 2 in males.

####### Material examined.

Holotype: ♂ (4.9 mm), NIBRIV0000753910. Geomeunyeo, Jeju-do Island, South Korea (33°14'23"N, 126°34'59"E), 30 Dec 2012, grab sampler (about 24 m depth), by Prof. HY Soh. Paratypes: 5 ♂♂ (3.2–4.5 mm) and 1 ♀ (3.1 mm), NIBRIV0000848928. Same data as holotype.

####### Diagnosis.

Gnathopod 1 elongate; coxa produced anterioventrally; basis 1.2× as long as coxa; merus half as long as basis; carpus elongate, 0.9× as long as basis; propodus half as long as carpus. Gnathopod 2 basis lateral borders forming developed lobe anteriodistally (not sac-like), with oblique stridulated ridges on the surface; carpus slightly elongate but shorter than that of gnathopod 1, 0.6× as long as basis; propodus stout, subovoid, 1.2× as long as carpus, palm defined by one acute spine. Female gnathopod 1 not elongate. Female gnathopod 2 as long as female gnathopod 1, but stouter; coxa anterioventral production weaker than that of male; basis with oblique stridulated ridges on lateral surface; merus 0.4× as long as basis; carpus 0.4× as long as basis; propodus without defining spine, but bearing defining robust seta medially. Pereopod 3 coxa with stridulated ridges on the surface along the ventral margin laterally in both sexes. Uropod 3 outer ramus biarticulated, 2^nd^ article vestigial; inner ramus scale-like, 0.2× as long as outer ramus.

####### Description.

***Holotype male.*** Head (Fig. 14A) 0.8× as long as pereonite 1; lateral cephalic lobe subtriangular; eye small, circular; antennal sinus deep.

Antenna 1 (Fig. 14B) peduncle 1^st^ article stout, 0.7× as long as head; 2^nd^ article slender, 1.3× as long as 1^st^ article; 3^rd^ article 0.8× as long as 2^nd^ article; accessory flagellum absent; flagellum 0.6× as long as peduncle 1^st^–3^rd^ articles combined, composed of seven articles (terminal article rudimentary).

Antenna 2 (Fig. 14C) 1.2× as long as antenna 1; peduncle 3^rd^ article exceeding end of cephalic lobe, 0.4× as long as 4^th^ article; 4^th^ article 1.1× as long as 2^nd^ article of antenna 1; 5^th^ article 0.9× as long as 4^th^ article; flagellum 0.6× as long as peduncle 3^rd^–5^th^ articles combined, composed of six articles (terminal article rudimentary).

Upper lip (Fig. 14D) convex anteriorly, entire, covered with minute setae.

Mandibles (Fig. 14E, F) with 5-dentate incisor, 4-dentate lacinia mobilis, and five raker setae on left mandible; with 6-dentate incisor, minutely dentate lacinia mobilis, and three raker setae on right mandible; molar well developed, triturative, with seven setae along the distal margin of right mandible; palp asymmetrical, composed of three articles, 3^rd^ article distally rounded, 0.8× as long as 2^nd^ article, with setae extending along most of posteriodistal margin.

Maxilla 1 (Fig. 14G, H) outer lobe with nine dentate robust setae on apical margin; palp biarticulated, distal article curved, a little swollen, with five setae on apical margin.

Maxilla 2 (Fig. 14I) inner lobe with an oblique row of plumose setae on surface; outer lobe longer and slightly dilated distally than inner lobe.

Maxilliped (Fig. 15A) inner lobe subrectangular, slightly expanded distally, with three nodular setae apically and one medial nodular seta subdistally; outer lobe exceeding half of palp 2^nd^ article, lined with eight robust setae along apex to medial margin; palp composed of four articles, 3^rd^ article slightly expanded distally, half as long as 2^nd^ article, 4^th^ article 0.6× as long as 3^rd^ article, with elongate seta apically (1.1× as long as 4^th^ article).

Gnathopod 1 (Fig. 15B, C) elongate; coxa subtriangular, half as wide as long, tapering and produced anterioventrally; ventral margin evenly rounded with six setae; basis subtrapezoidal, 1.2× as long as coxa, anterior margin lateral border lobate distally, with three plumose setae subproximally, posterior margin convex, more swollen at the middle, with six elongate setae; merus half as long as basis; carpus elongate, 0.9× as long as basis, 0.3× as wide as long, slightly widened distally, carpal lobe not developed; propodus half as long as carpus, rounded anteriorly, palm half as long as posterior margin, serrated irregularly, slightly concave, defined by one robust seta medially; dactylus half as long as propodus, with five teeth (proximal three minute) on inner margin.

Gnathopod 2 (Fig. 15D) stout, 0.7× as long as gnathopod 1; coxa subtrapezoidal, 1.4× as long as that of gnathopod 1, 0.4× as wide as long, produced anterioventrally; basis 0.7× as long as coxa, 0.4× as wide as long, anterior margin lateral borders forming developed lobe (but not sac-like) distally bearing oblique stridulated ridges on surface, posterior margin convex, with three elongate setae at the middle; merus 0.6× as long as basis; carpus slightly elongate but shorter than that of gnathopod 1, 0.6× as long as basis, with moderate carpal lobe posteriorly; propodus stout, subovoid, 1.2× as long as carpus, anterior margin evenly rounded, posterior margin 0.7× as long as anterior margin, palm oblique, half as long as posterior margin, defined by one acute spine; dactylus 0.4× as long as propodus, with two teeth on inner margin.

Pereopod 3 (Fig. 15E) coxa widened ventrally, somewhat curved, 0.9× as long as that of gnathopod 2, 0.7× as wide as long, with stridulated ridges on surface along ventral margin laterally; basis expanded posteriorly, 0.4× as wide as long, posterior margin convex, with four plumose setae in the middle; merus 0.7× as long and 0.6× as wide as basis, anterior margin weakly expanded, with twelve plumose setae submarginally, distal corner weakly produced; posterior margin not expanded, with four plumose setae; carpus half as long as merus; propodus diminished distally, half as long as basis; dactylus half as long as propodus.

Pereopod 4 (Fig. 16A) coxa not widened distally, slightly curved, half as wide as long, without stridulated ridges; other articles similar to those of pereopod 3 in shape, but plumose setae different in position.

Pereopod 5 (Fig. 16B) coxa 0.9× as long as that of pereopod 4, bilobed, anterior lobe subovoid, expanded ventrally, posterior lobe small, extended backwards; basis subovoid, broad, more expanded proximally, as long as wide, with four plumose setae on medial surface anterioproximally; merus subrectangular, a little lobate anteriodistally, 0.4× as long as basis, half as wide as long; carpus rectangular, as long and 0.9× as wide as merus; propodus 0.9× as long as carpus, with a pair of distal locking setae unequal in length (longer seta reaching end of dactylus), with a group of three setae (longest seta 0.9× as long as propodus) at anteriodistal corner; dactylus short, armed with one accessory cusp on outer margin.

Pereopod 6 (Fig. 16C) as long as, but slender than pereopod 5; coxa bilobed, anterior lobe small, posterior lobe dilated posterioventrally; basis subovoid, 0.7× as wide and as that of pereopod 5, anterior margin convex, slightly dilated distally, posterior margin convex, with four notches irregularly; merus rectangular, weakly widened distally, half as long as basis, 0.3× as wide as long; carpus as long as merus; propodus as long as carpus, with a pair of distal locking setae unequal in length, with a group of five setae (longest seta 0.8× as long as propodus) at anteriodistal corner; dactylus half as long as propodus, without accessory cusp on outer margin.

Pereopod 7 (Fig. 16D) as long as pereopod 6; coxa unilobed; basis subovoid, as long and 0.8× as wide as that of pereopod 6, 0.6× as wide as long, anterior margin rather convex, posterior margin weakly dilated distally; posterior margin proximal extension weak, with five notches irregularly; merus rectangular, linear, 0.2× as wide as long, half as long as basis; carpus as long as merus; propodus 1.1× as long as carpus, with a pair of distal locking setae unequal in length shorter than that of pereopods 5–6, with a group of more than five setae at posteriodistal corner; dactylus half as long as propodus, armed with one accessory cusp on outer margin.

Epimera 2 and 3 (Fig. 16E) each posterioventral corner produced backwards.

Uropod 1 (Fig. 16F) peduncle without distoventral spine, with seven robust setae on dorsolateral margin and one robust seta distally on dorsomedial margin; both rami 0.7× as long as peduncle, with three and two robust setae on dorsal margin of outer and inner rami, each apex with one stout seta and two robust setae.

Uropod 2 (Fig. 16G) 0.6× as long as uropod 1; peduncle half as long as that of uropod 1, with three robust setae on dorsolateral margin and one robust seta distally on dorsomedial margin; inner ramus 1.2× as long as peduncle, with three robust setae on dorsal margin and with one stout seta and two robust setae at apex; outer ramus 0.8× as long as inner ramus, with two robust setae on dorsal margin and with one stout seta and two robust setae at apex.

Uropod 3 (Fig. 16H) as long as uropod 2; peduncle 1.4× as long as that of uropod 2; outer ramus biarticulated, 0.6× as long as peduncle, 2^nd^ article vestigial, with two elongate setae subapically; inner ramus scale-like, 0.2× as long as outer ramus.

Telson (Fig. 16I) triangular in dorsal view, with a pair of simple setae and a pair of sensory setae on each side.

***Paratype female.*** Gnathopod 1 (Fig. 17B, C) not elongate; coxa similar to that of male; basis subtrapezoidal, posterior margin flatter than that of male; carpus not elongate, 0.6× as long and 1.1× as wide as basis, carpal lobe blunt; propodus as long as carpus, posterior margin convex, palm serrated, slightly convex proximally and bearing weak cavity distally, defined by one robust seta medially; dactylus 0.6× as long as propodus, inner margin serrated, with one tooth.

Gnathopod 2 (Fig. 17D, E) as long as but stouter than gnathopod 1; coxa subrectangular, 1.2× as long as that of gnathopod 1, half as wide as long, anterioventral production weaker than that of male, oostegite 1.3× as long as basis, 0.4× as wide as long; basis anterior margin lateral borders forming well-developed lobe distally bearing oblique stridulated ridges on surface; merus 0.4× as long as basis; carpus 0.4× as long as basis, with developed carpal lobe posteriorly; propodus stout, 0.6× as long as basis, posterior margin 0.6× as long as anterior margin, palm oblique, 0.9× as long as posterior margin, without defining spine, but bearing defining robust seta medially; dactylus 0.4× as long as propodus, inner margin serrated, with one tooth.

Pereopod 3 (Fig. 17F) coxa widened distally, 0.6× as wide as long, slightly produced posterioventrally, with stridulated ridges on surface along ventral margin, oostegite as long and as wide as that of gnathopod 2; basis 0.4× as wide as long, anterior margin slightly expanded proximally, posterior margin evenly rounded; merus half as wide as long, anterior margin expanded, with eleven plumose setae submarginally, distal corner weakly produced; posterior margin not expanded, with four plumose setae; carpus rectangular, half as long as merus; propodus diminished distally, 0.4× as long as basis; dactylus half as long as propodus.

Oostegites on gnathopod 2 as long as that of pereopod 3. That of pereopod 5 0.7× as long as that of pereopod 4 (Fig. 17D, F–H).

####### Remarks.

This species is closely related to *Photis japonica* Hirayama, 1984 in sharing a significantly elongate gnathopod 1 in mature males, but differs in having stridulated ridges on the lateral surface of gnathopod 1 basis and pereopod 3 coxa, which are arranged along the ventral margin in both sexes. Also, *Photis longicarpus* sp. nov. has a defining spine on gnathopod 2 palm in males only, but *P. japonica* has it in both sexes. Moreover, the uropod 3 outer ramus of *P. longicarpus* sp. nov. is biarticulated (distal article is vestigial), but that of *P. japonica* is uniarticulated (Hirayama 1984).

###### Photis posterolobus sp. nov.

Taxon classificationAnimaliaAmphipodaPhotidae

D5F8D493-F9B6-5AB0-9006-8F5A33DA2F3B

http://zoobank.org/3C32E5BE-E031-4B56-A37E-0E9669F08972

Figs 18 , 19 , 20

####### Etymology.

The composite epithet of the specific name, *posterolobus*, is a combination of the Latin words *posterus* and *lobus*, referring to the presence of a posterior lobe produced distally on the ischium of male gnathopod 2.

####### Material examined.

Holotype: ♂ (4.8 mm), NIBRIV0000753909. Geomeunyeo, Jeju-do Island, South Korea (33°14'23"N, 126°34'59"E), 24 Dec 2012, grab sampler (about 24 m depth), by Prof. HY Soh.

####### Diagnosis.

Male gnathopod 1 palmar margin weakly sinuated. Male gnathopod 2 basis lateral border forming a well-developed sac-like lobe; ischium posterior margin with lateral lobe acutely produced distally; merus rectangular, stout, half as long as basis, with transparent lobe distally; carpus anterior margin irregular, carpal lobe well developed; propodus stout, as long as basis, posterior margin with one elongate process half as long as anterior margin, palmar margin oblique, 0.7× as long as anterior margin, with two spines (proximal larger than distal), without defining seta. Stridulated ridges only present on gnathopod 2 basis and coxae 3 and 4 in males (unclear in females).

####### Description.

**Figure 18:**
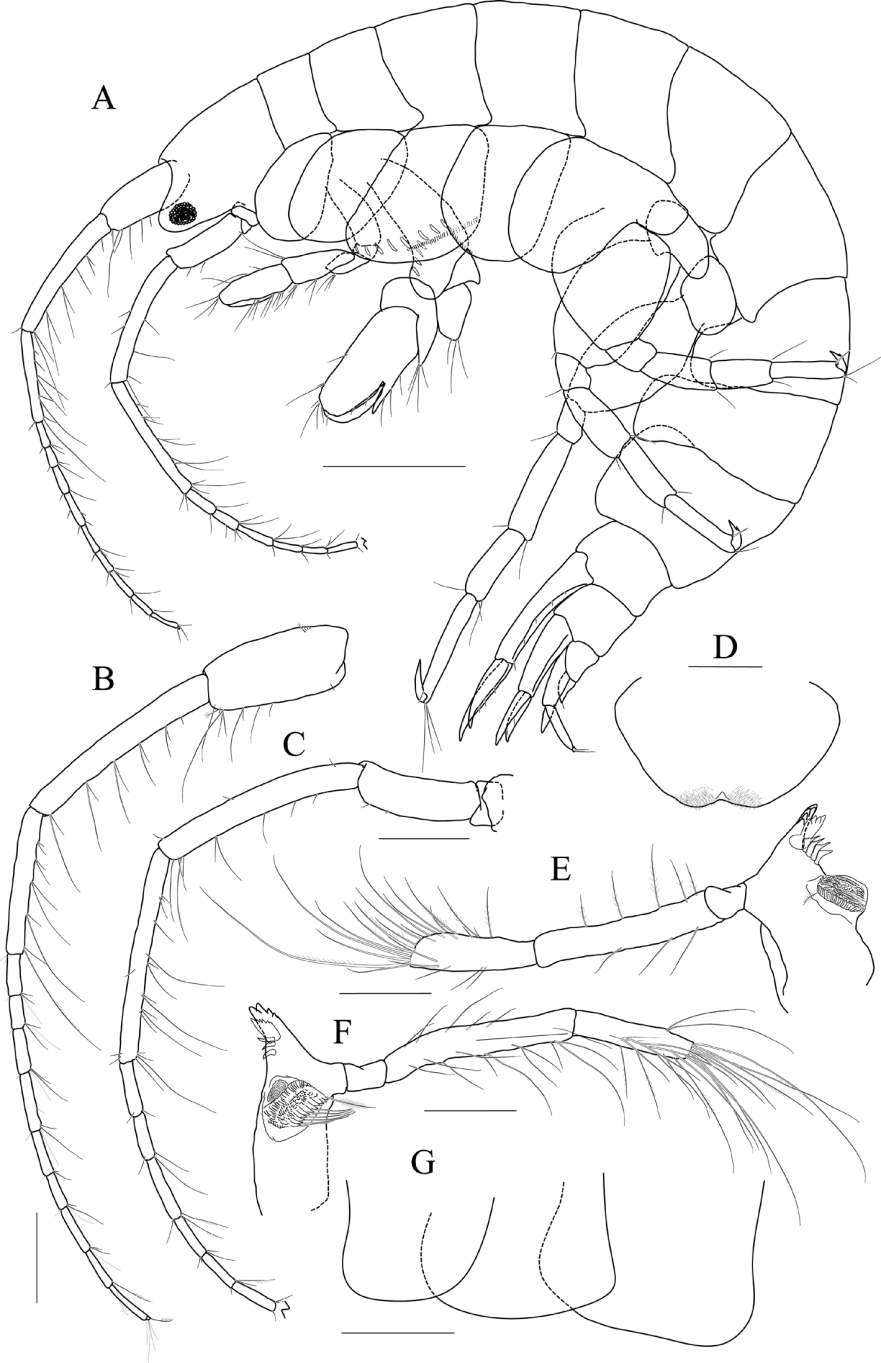
*Photis posterolobus* sp. nov., holotype, NIBRIV0000753909, male, 4.8 mm, Geomeunyeo, Jeju-do, South Korea. **A** habitus **B** antenna 1 **C** antenna 2 **D** upper lip **E** left mandible **F** right mandible **G** pleonal epimera. Scale bars: 0.05 mm (**D**), 0.1 mm (**E, F**), 0.2 mm (**B, C, G**), 0.5 mm (**A**).

***Holotype male.*** Head (Fig. 18A) as long as pereonites 1 and 2 combined; lateral cephalic lobe rounded; eye circular, moderate in size; located in the middle of lateral lobe; antennal sinus deep.

Antenna 1 (Fig. 18B) 0.4× as long as body; peduncle 1^st^ article stout, 0.7× as long as head; 2^nd^ article slender, 1.4× as long as 1^st^ article; 3^rd^ article 0.7× as long as 2^nd^ article; accessory flagellum absent; flagellum 0.8× as long as peduncle 1^st^–3^rd^ articles combined, composed of ten articles (terminal article rudimentary).

Antenna 2 (Fig. 18C) peduncle 3^th^ article exceeding end of lateral cephalic lobe; 4^th^ and 5^th^ articles as long as 2^nd^ article; flagellum 0.6× as long as peduncle 3^rd^–5^th^ articles combined, composed of more than six articles.

Upper lip (Fig. 18D) convex anteriorly, with notch in the middle, covered with minute setae.

Mandibles (Fig. 18E, F) with 5-dentate incisor, 4-dentate lacinia mobilis, and four raker setae on left mandible; with 1/2 and 5-dentate incisor, minutely dentate lacinia mobilis, and three raker setae on right mandible; molar well developed, triturative, with seven setae along the distal margin of right mandible; palp asymmetrical, composed of three articles, 3^rd^ article distally rounded, 0.7× as long as 2^nd^ article, with setae extending along most of posteriodistal margin.

**Figure 19:**
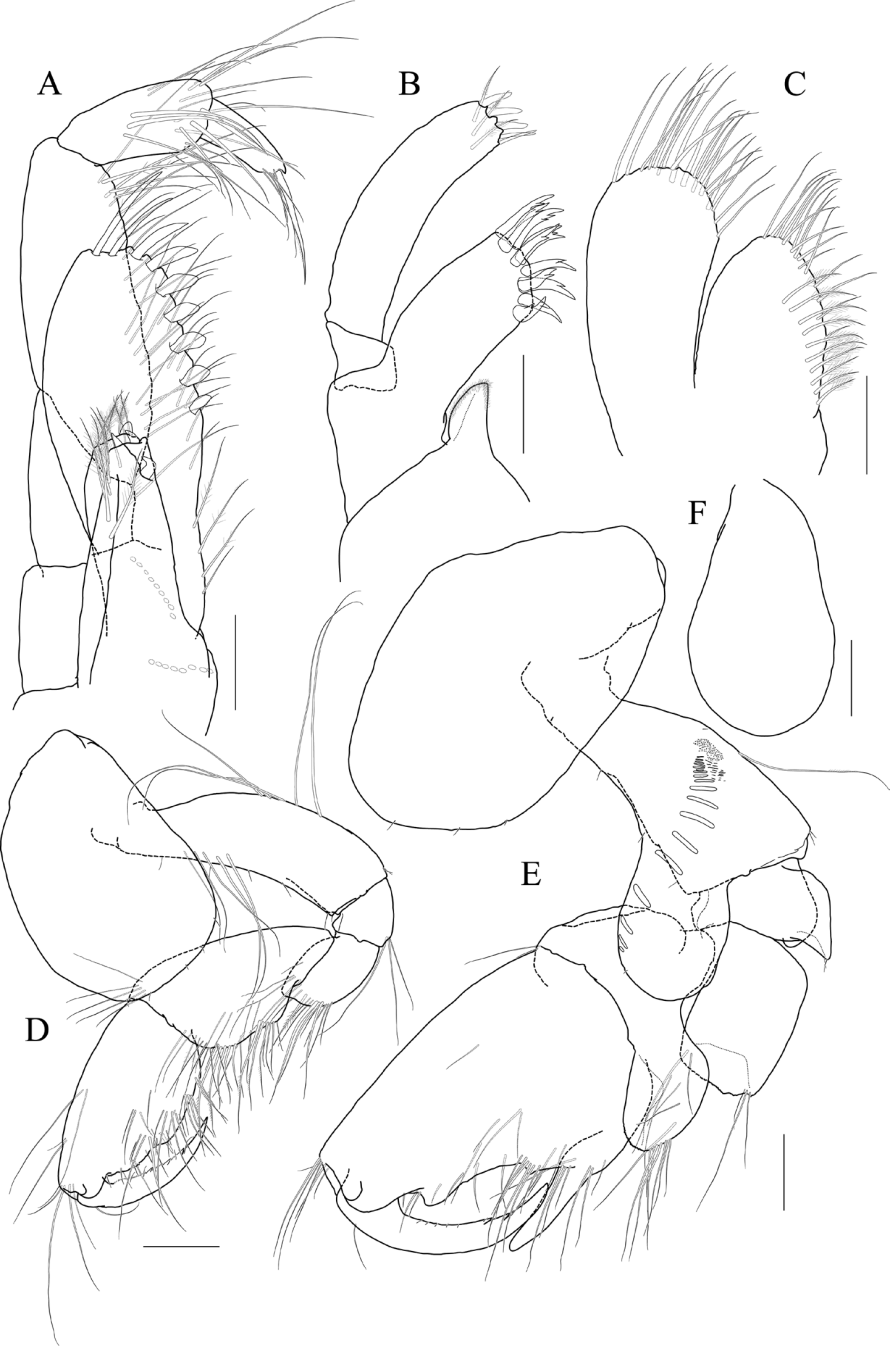
*Photis posterolobus* sp. nov., holotype, NIBRIV0000753909, male, 4.8 mm, Geomeunyeo, Jeju-do, South Korea. **A** maxilliped **B** maxilla 1 **C** maxilla 2 **D** gnathopod 1 **E** gnathopod 2 **F** gill of gnathopod 2. Scale bars: 0.05 mm (**A–C**), 0.1 mm (**D–F**).

Maxilliped (Fig. 19A) inner lobe subrectangular, weakly expanded distally, with three nodular setae apically and one medial nodular seta subdistally; outer lobe exceeding half of palp 2^nd^ article, lined with nine robust setae along apex to medial margin; palp composed of four articles, 3^rd^ article slightly expanded distally, 0.4× as long as 2^nd^ article, 4^th^ article 0.8× as long as 3^rd^ article, with elongate seta apically (1.2× as long as 4^th^ article).

Maxilla 1 (Fig. 19B) inner lobe small, covered with minute setae; outer lobe with ten dentate robust setae on apical margin; palp biarticulated, distal article curved, with five setae on apical margin.

Maxilla 2 (Fig. 19C) inner lobe with an oblique row of plumose setae on surface; outer lobe longer and slightly dilated distally than inner lobe.

Gnathopod 1 (Fig. 19D) coxa 0.7× as wide as long, evenly rounded anterioventrally, slightly expanded anteriorly; basis as long as coxa, anterior margin lateral border forming weak lobe distally, with five elongate setae subproximally, posterior margin convex, with five elongate setae at the middle; carpus subtrapezoidal, 0.8× as long as basis, half as wide as long, posterior lobe blunt; propodus as long and wide as carpus, rounded anteriorly, with minute serrations irregularly along palm and posterior margin, palm 0.8× as long as posterior margin, weakly bisinuate, defined by one robust seta medially; dactylus 0.7× as long as propodus, exceeding palm, inner margin serrated, with three teeth.

Gnathopod 2 (Fig. 19E, F) stout, coxa subrectangular, 0.8× as wide as long, produced anterioventrally; basis anterior margin lateral border forming well-developed lobe distally (sac-like lobe reaching middle of carpus) bearing oblique stridulated ridges on surface; ischium anterior lobe small, posterior margin lateral border forming lobe produced distally; merus rectangular, half as long as basis, with transparent lobe distally; carpus anterior margin irregular, carpal lobe well developed; propodus stout, as long as basis, posterior margin with one elongate process half as long as anterior margin, palmar margin oblique, 0.7× as long as anterior margin, with two spines (proximal larger than distal), without defining seta; dactylus 0.7× as long as propodus, with two teeth on inner margin.

**Figure 20:**
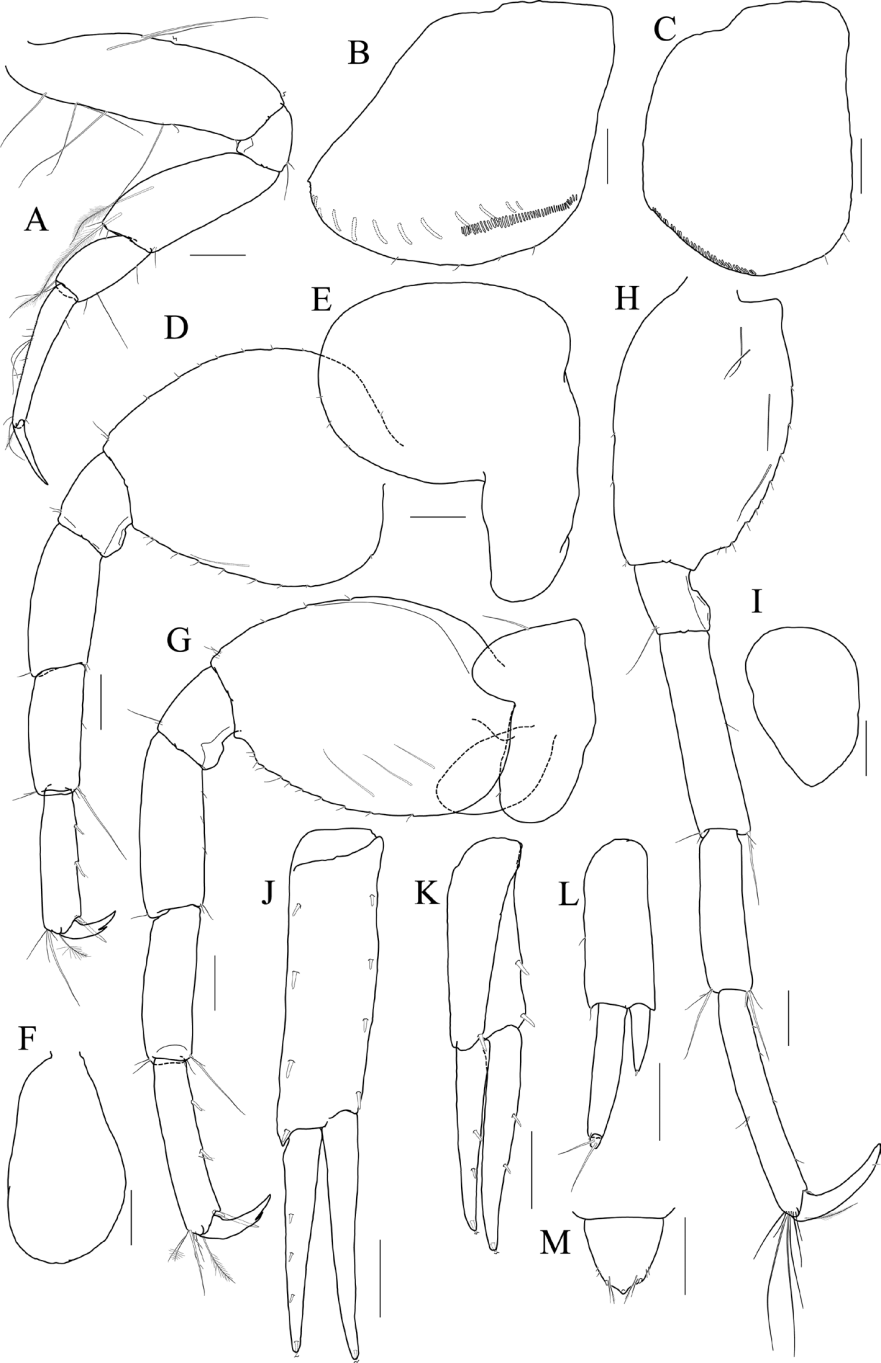
*Photis posterolobus* sp. nov., holotype, NIBRIV0000753909, male, 4.8 mm, Geomeunyeo, Jeju-do, South Korea. **A** coxa 3 **B** pereopod 3 **C** coxa 4 **D** coxa 5 **E** pereopod 5 **F** gill of pereopod 5 **G** pereopod 6 **H** coxa 7 **I** pereopod 7 **J** uropod 1 **K** uropod 2 **L** uropod 3 **M** telson. Scale bars: 0.1 mm.

Pereopod 3 (Fig. 20A, B) coxa produced anterioventrally, as long as wide, with stridulated ridges on medial surface and short stridulated ridges near the posterioventral corner on lateral surface submarginally; basis 0.3× as wide as long, posterior margin expanded; merus 0.6× as long as basis, anterior margin expanded distally, with two plumose setae submarginally, distal corner weakly produced; carpus half as long as merus, evenly rounded anteriorly; propodus slender, diminished distally, 0.6× as long as basis; dactylus half as long as propodus.

Pereopod 4 coxa (Fig. 20C) not widened distally, as long as that of pereopod 3, with stridulated ridges along anterioventral corner oblique.

Pereopod 5 (Fig. 20D–F) coxa bilobed, large, anterior lobe subovoid, expanded ventrally, posterior lobe small, expanded backwards; basis subovoid, broad, more expanded proximally, 0.8× as long as wide; merus subrectangular, slightly convex anteriorly, half as long as basis, 0.4× as wide as long; carpus 0.8× as long as merus; propodus 1.1× as long as carpus, with a pair of distal locking setae unequal in length (longer seta 0.8× as long as dactylus), with a group of four setae (longest seta half as long as propodus) at anteriodistal corner; dactylus half as long as propodus, armed with one accessory cusp on outer margin.

Pereopod 6 (Fig. 20G) 1.1× as long as pereopod 5; coxa bilobed, anterior lobe small, posterior lobe dilated posterioventrally; basis subovoid, 0.8× as wide as long, anterior margin convex, posterior margin slightly dilated proximally; merus 0.6× as long as basis, 0.3× as wide as long; carpus 0.8× as long as merus; propodus 1.2× as long as carpus, with a pair of distal locking setae unequal in length (longer seta 0.6× as long as dactylus), with a group of five setae (longest seta 0.8× as long as propodus) at anteriodistal corner; dactylus half as long as propodus, without accessory cusp on outer margin.

Pereopod 7 (Fig. 20H–I) 1.3× as long as pereopod 6; coxa unilobed, produced posteriorly; basis subovoid, 0.8× as wide as that of pereopod 6, 0.6× as wide as long, anterior margin rather convex, posterior margin with one blunt extension proximally; merus rectangular, 0.7× as long as basis; 0.2× as wide as long; carpus 0.8× as long as merus; propodus 1.5× as long as carpus, with a pair of distal locking setae unequal in length (smaller than those of pereopods 5 and 6), with a group of more than seven setae at posteriodistal corner; dactylus 0.4× as long as propodus, armed with one accessory cusp on outer margin.

Epimeron 1 slightly extended anterioventrally. Epimera 2 and 3 each posterioventral corner produced backwards, but not acute (Fig. 18G).

Uropod 1 (Fig. 20J) peduncle without distoventral spine, with four robust setae on both dorsolateral and dorsomedial margins; inner ramus 0.8× as long as peduncle, with one subapical seta only; outer ramus 0.9× as long as inner ramus, with three dorsolateral setae and one subapical seta.

Uropod 2 (Fig. 20K) 0.8× as long as uropod 1; peduncle 0.7× as long as that of uropod 1; inner ramus 1.1× as long as peduncle, with two dorsal robust setae and one subapical seta; outer ramus 0.9× as long as inner ramus, with two dorsal robust setae and one subapical seta.

Uropod 3 (Fig. 20L) 0.8× as long as uropod 2; peduncle 0.8× as long as that of uropod 2; outer ramus biarticulated, 0.9× as long as peduncle, last article vestigial, with two elongate setae subapically; inner ramus scale-like, 0.4× as long as outer ramus.

Telson (Fig. 20M) subtriangular in dorsal view, with a pair of simple setae, a pair of sensory setae, and one nodular robust seta on each side.

####### Remarks.

*Photis posterolobus* sp. nov. is closely related to nine *Photis* species [*P. bronca* sp. nov.; *P. fischmanni* Gurjanova, 1951; *P. guerrai* Tato & Moreira, 2017; *P. hawaiensis* JL Barnard, 1955; *P. kapapa* JL Barnard, 1970; *P. lecroyae* Ortiz, Varela & Lalana, 2011; *P. longicaudata* (Spence Bate & Westwood, 1863); *P. sarae* Souza-Filho & Serejo, 2010; and *P. tenuicornis* GO Sars, 1882] in bearing a very well-developed sac-like lobe anteriodistally on the basis of male gnathopod 2. However, *Photis posterolobus* sp. nov. can be distinguished from above species by the presence of a posterior lobe produced distally in the ischium of male gnathopod 2 that is absent in the above species (Bate and Westwood 1863; Sars 1883; Gurjanova 1951, 1955; Barnard 1955, 1970; Ren 2006; Souza-Filho and Serejo 2010; Tato and Moreira 2017).

##### Genus *Podoceropsis* Boeck, 1861

###### Podoceropsis insinuomanus sp. nov.

Taxon classificationAnimaliaAmphipodaPhotidae

349D3C08-00D0-5C22-9518-13E60AA647DF

http://zoobank.org/9B529875-E9ED-428D-97B8-C39FDDABDD91

Figs 21 , 22 , 23

####### Etymology.

The composite epithet of the specific name, *insinuomanus*, is a combination of the Latin words *insinuo* and *manus*, meaning sinuous hand. This name refers to the strongly sinuated shape of male gnathopod 2 palmar margin.

####### Material examined.

Holotype: ♂ (4.2 mm), NIBRIV0000806531. Gageo-do Island, Jeollanam-do, South Korea (34°02'57"N, 125°08'14"E), 28 Oct 2015, light trap (6.6 m depth), by TW Jung.

Additional material: ♂, 3.3 mm, NIBRIV0000848933. Daryeo-do Island, Bukchon, Jeju-do, South Korea (34°34'56"N, 126°57'17"E), 30 Nov 2012, grab sampler (about 20 m depth), by Prof. HY Soh.

####### Diagnosis.

Gnathopod 2 propodus stout, subovoid, 1.6× as long as basis, palmar margin strongly bisinuate, wrinkly, with subrectangular protrusion near dactylus base and excavated posteriodistally, defined by one robust seta medially. Pereopod 5 basis subovoid, posteriodistal notch really weak.

####### Description.

**Figure 21:**
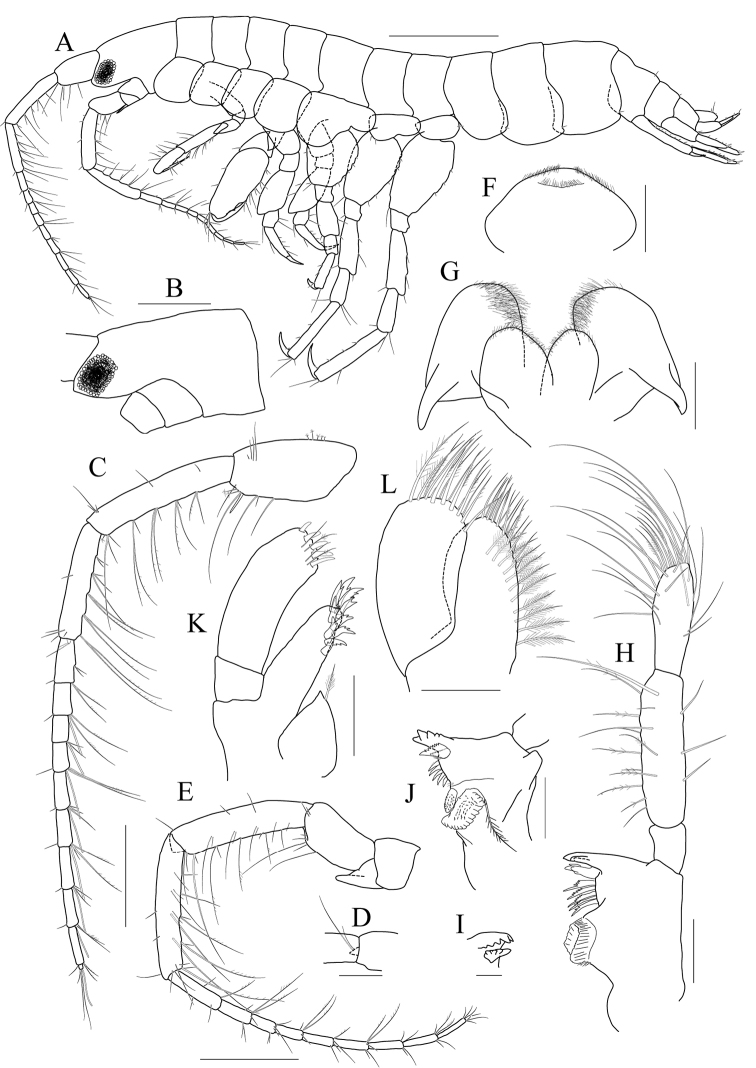
*Podoceropsis insinuomanus* sp. nov., holotype, NIBRIV0000806531, male, 4.2 mm, Gageo-do, South Korea. **A** habitus **B** head **C** antenna 1 **D** accessory flagellum **E** antenna 2 **F** upper lip **G** lower lip **H, I** left mandible **J** right mandible **K** maxilla 1 **L** maxilla 2. Scale bars: 0.02 mm (**I**), 0.05 mm (**F–H, J–L**), 0.2 mm (**C, E**), 0.5 mm (**A, B**).

***Holotype male.*** Head (Fig. 21B) 1.2× as long as pereonites 1–2 combined; lateral cephalic lobe subtriangular, with small tip additionally on apex; eye circular, large, occupying most of lateral cephalic lobe; antennal sinus deep.

Antenna 1 (Fig. 21C, D) peduncle 1^st^ article stout, half as long as head, with one robust seta at posteriodistal corner; 2^nd^ article 1.6× as long as 1^st^ article; 3^rd^ article 0.7× as long as 2^nd^ article; accessory flagellum uniarticulated, vestigial; flagellum 0.9× as long as peduncle 1^st^–3^rd^ articles combined, composed of ten articles (terminal article rudimentary).

Antenna 2 (Fig. 21E) as long as antenna 1; each peduncle’s 4^th^ and 5^th^ articles as long as 2^nd^ article of antenna 1; flagellum 0.9× as long as peduncle 3^rd^–5^th^ articles combined, composed of ten articles (terminal article rudimentary).

Upper lip (Fig. 21F) convex anteriorly, entire, covered with minute setae.

Lower lip (Fig. 21G) inner lobe subovoid, outer lobe apex rounded, covered with minute setae, with one robust seta at mediodistal corner; mandibular process well developed.

Mandibles (Fig. 21H–J) with 1/2 and 4-dentate incisor, 4-dentate lacinia mobilis, and five raker setae on left mandible; with 5-dentate incisor, minutely dentate lacinia mobilis, and five raker setae on right mandible; molar well developed, triturative; palp asymmetrical, composed of three articles, 3^rd^ article distally rounded, 0.7× as long as 2^nd^ article, with setae extending along most of posteriodistal margin.

Maxilla 1 (Fig. 21K) inner lobe small, produced distally, with one plumose seta; outer lobe with ten dentate robust setae on apical margin; palp biarticulated, distal article curved, not swollen, with five setae on apical margin.

Maxilla 2 (Fig. 21L) inner lobe with an oblique row of plumose setae on surface; outer lobe slightly larger than inner lobe.

**Figure 22:**
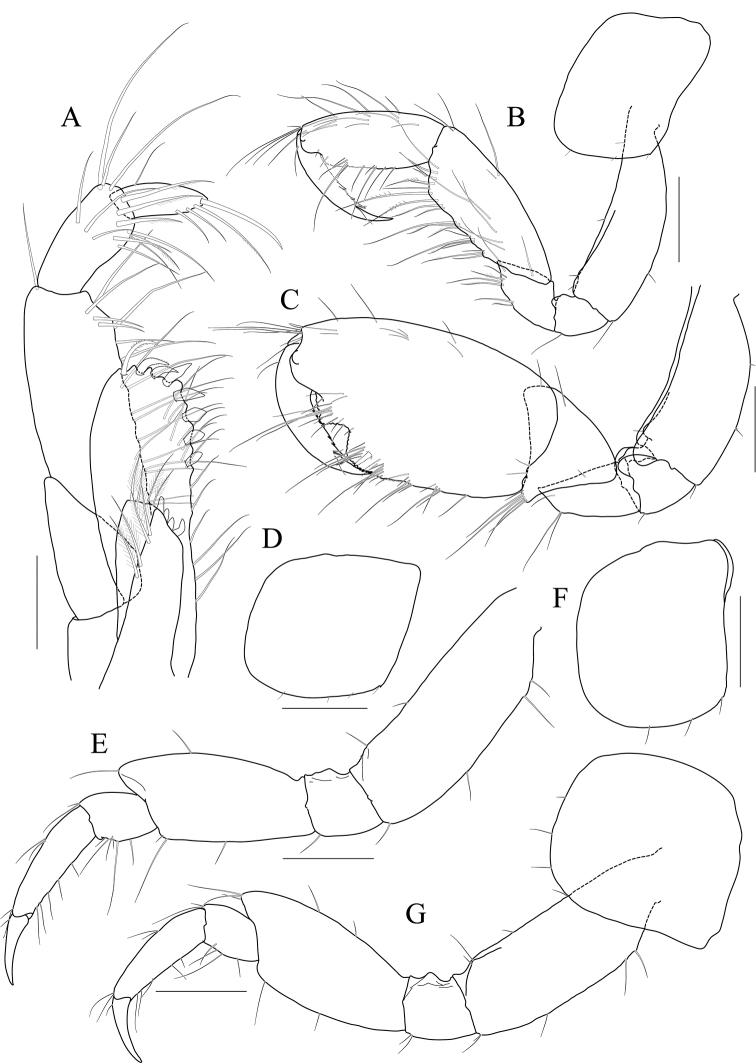
*Podoceropsis insinuomanus* sp. nov., holotype, NIBRIV0000806531, male, 4.2 mm, Gageo-do Island, South Korea. **A** maxilliped **B** gnathopod 1 **C** coxa 2 **D** gnathopod 2 **E** coxa 3 **F** pereopod 3 **G** pereopod 4. Scale bars: 0.05 mm (**A**), 0.1 mm (**B–G**).

Maxilliped (Fig. 22A) inner lobe subrectangular, slightly expanded distally, with three nodular setae apically and one medial nodular seta subdistally; outer lobe exceeding half of palp 2^nd^ article, lined with eight robust setae along apex to medial margin; palp composed of four articles, 3^rd^ article slightly expanded distally, 0.4× as long as 2^nd^ article, 4^th^ article 0.8× as long as 3^rd^ article, with elongate seta apically (as long as 4^th^ article).

Gnathopod 1 (Fig. 22B) coxa subrhomboid, 0.8× as wide as long; basis subtrapezoidal, 1.3× as long as coxa, scarcely setose, anterior margin lateral border weakly lobate distally, posterior margin rather convex; carpus elongate, as long as basis, 0.3 as wide as long, slightly widened distally, carpal lobe not developed; propodus 0.7× as long as carpus, rounded anteriorly, palm indistinct, posterior margin minutely serrated on distal half margin; dactylus elongate, 0.9× as long as propodus, with three teeth on inner margin.

Gnathopod 2 (Fig. 22C, D) stout, 1.1× as long as gnathopod 1; coxa subrhomboid, as long as and 0.8× as wide as that of gnathopod 1, rounded anterioventrally; basis scarcely setose, anterior margin both medial and lateral borders forming lobes distally but that of lateral more produced distally; ischium anterior margin with both lateral and medial lobes well developed; carpus with weak carpal lobe posteriorly; propodus stout, subovoid, 1.6× as long as basis, 0.6× as wide as long, anterior margin evenly rounded, posterior margin 0.6× as long as anterior margin, palmar margin oblique, strongly bisinuate, wrinkly, with subrectangular protrusion near dactylus base and excavated posteriodistally, defined by one robust seta medially; dactylus half as long as propodus, slightly exceeding palm, with five teeth on inner margin.

Pereopod 3 (Fig. 22E, F) coxa quadrate, 1.2× as long as that of gnathopod 2, evenly rounded anterioventrally; basis subtrapezoidal, expanded posteriorly, 0.4× as wide as long; merus 0.8× as long as basis, as wide as basis, expanded anteriodistally, produced distal corner reaching half of carpus; carpus almost rectangular, short, 0.3× as long as basis; propodus diminished distally, half as long as basis; dactylus 0.6× as long as propodus.

Pereopod 4 (Fig. 22G) coxa 1.2× as wide as that of pereopod 3; other articles almost similar to those of pereopod 3.

**Figure 23:**
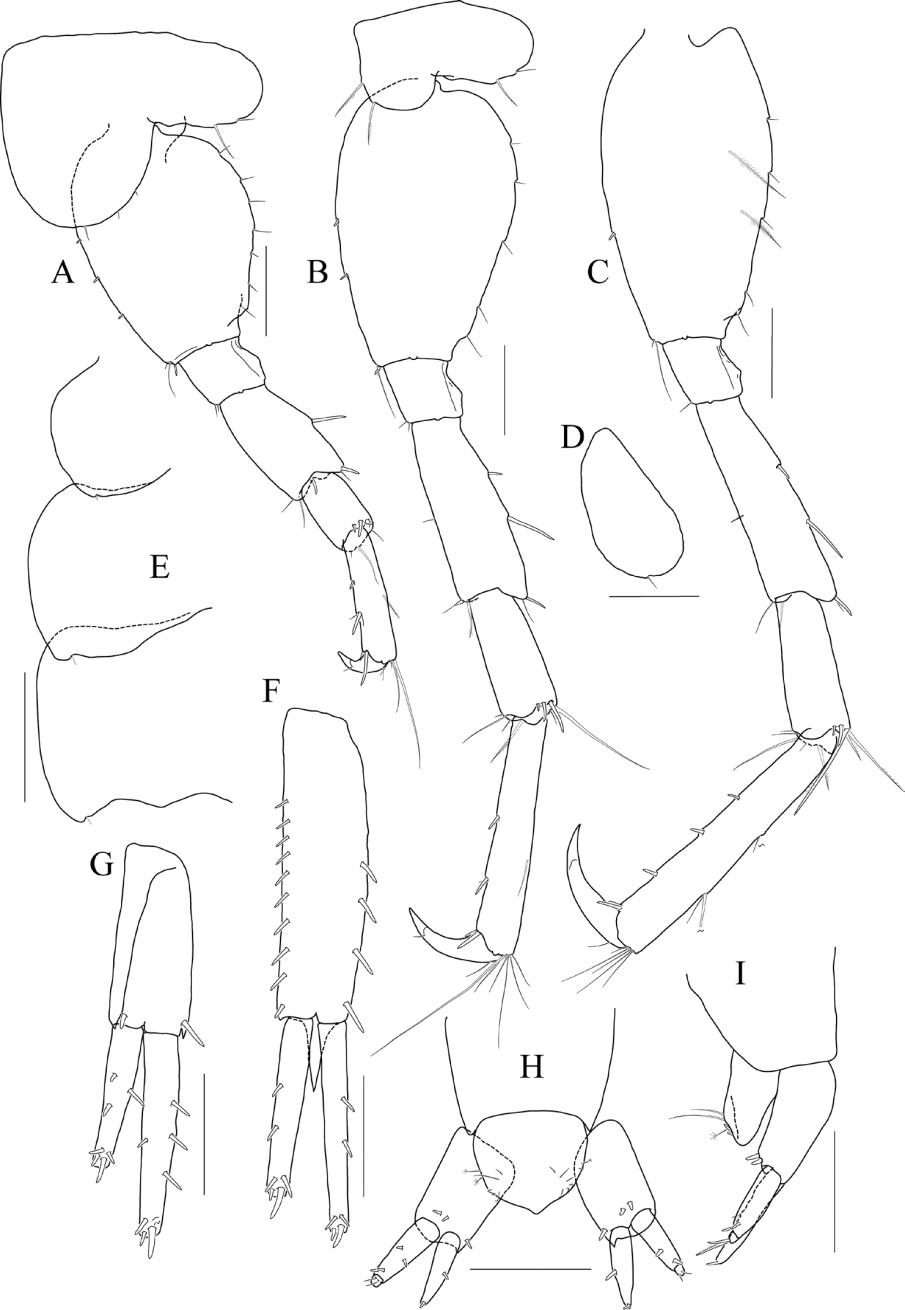
*Podoceropsis insinuomanus* sp. nov., holotype, NIBRIV0000806531, male, 4.2 mm, Gageo-do Island, South Korea. **A** pereopod 5 **B** pereopod 6 **C** coxa 7 **D** pereopod 7 **E** pleonal epimera **F** uropod 1 **G** uropod 2 **H, I** uropod 3 and telson, dorsal (**H**) and lateral (**I**). Scale bars: 0.1 mm (**A–D, F–I**), 0.2 mm (**E**).

Pereopod 5 (Fig. 23A) coxa bilobed, anterior lobe rounded and expanded ventrally, 0.9× as wide as basis, posterior lobe narrow, extended backwards, with one robust and one simple seta; basis subovoid, broader proximally, 0.7× as wide as long, anterior margin rounded, posterior margin weakly crenulated, lined with simple setae only, with weak distal notch; merus slightly widened distally, half as long as basis, 0.6× as wide as long, not twisted, posterior margin with one elongate robust seta at middle; carpus almost rectangular, slightly plump, 0.7× as long as merus, 0.6× as wide as long; propodus 1.6× as long as carpus, somewhat widened distally, with two robust setae on anterior margin and one pair of unequal locking setae distally (longer seta exceeding dactylus); dactylus falcate. 0.4× as long as propodus.

Pereopod 6 (Fig. 23B) 1.4× as long as pereopod 5; coxa bilobed, anterior lobe smaller than that of pereopod 5, with two setae anteriorly, posterior lobe slightly larger than anterior lobe, weakly dilated posterioventrally, with two setae posteriorly; basis subovoid, broader proximally, as wide and 1.3× as long as that of pereopod 5, anterior margin evenly rounded, with two robust setae, posterior margin weakly crenulated, lined with simple setae only, with weak distal notch; merus 0.6× as long as basis, 0.4× as wide as long, posterior margin expanded distally, with elongate robust setae (longest seta 0.3× as long as merus); carpus subrectangular, slightly widened distally, 0.7× as long as merus, 0.4× as wide as long; propodus linear, 1.7× as long as carpus, a little widened distally, with a pair of unequal locking setae; dactylus stout, falcate, half as long as propodus.

Pereopod 7 (Fig. 23C, D) 1.2× as long as pereopod 6; coxa unilobed, dilated posterioventrally; basis subovoid, as wide and 1.1× as long as that of pereopod 6, anterior margin evenly rounded, with one robust seta, posterior margin weakly crenulated, lined with simple setae only, proximal cusp developed proximally, with weak distal notch, medial surface with two plumose setae, merus expanded posteriodistally, 1.2× as long and 0.9× as wide as that of pereopod 6, posterior margin with elongate robust setae (longest seta 0.3× as long as merus); carpus subrectangular, slightly widened distally, 0.7× as long as merus, with a group of robust setae at posteriodistal corner (longest seta 0.6× as long as carpus); propodus as long as basis, slightly widened distally, distal locking setae unequal in length; dactylus stout, 0.4× as long as propodus.

Epimeron 1 rounded, with a notch bearing minute seta on posterioventral corner. Epimera 2 and 3 each posterioventral corner produced backwards, with a notch bearing minute seta (Fig. 23E).

Uropod 1 (Fig. 23F) peduncle with distoventral spine 0.2× as long as peduncle, with four dorsomedial and ten dorsolateral robust setae; inner ramus 0.7× as long as peduncle, with two dorsomedial robust setae and one group of four robust setae on apex; outer ramus 0.8× as long as inner ramus, with two dorsolateral robust setae and one group of four robust setae on apex.

Uropod 2 (Fig. 23G) 0.8× as long as uropod 1; peduncle 0.6× as long as that of uropod 1, both dorsal margins with distal robust seta only; inner ramus 1.1× as long as peduncle, with three dorsomedial robust setae and one dorsolateral robust seta, with one group of four robust setae on apex; outer ramus 0.7× as long as inner ramus, with one dorsomedial robust seta and two dorsolateral robust setae, with one group of four robust setae on apex.

Uropod 3 (Fig. 23H, I) 0.4× as long as uropod 1; peduncle 0.6× as long as that of uropod 2; inner ramus 0.7× as long as peduncle, tapering distally, terminated by one robust seta; outer ramus as long as inner ramus, biarticulated, 2^nd^ article vestigial, with two elongate setae subapically.

Telson (Fig. 23H, I) subtrapezoidal in dorsal view, produced in apex, margins rounded, with a pair of robust setae on each side.

####### Remarks.

*Podoceropsis insinuomanus* sp. nov. is closely related to *Podoceropsis barnardi* Kudryashov & Tzvetkova, 1975, reported from the southern and western Sakhalin, eastern Russia (Kudryashov and Tzvetkova 1975) and Vancouver, Canada (Conlan 1983), in the similar shape of the palmar margin of male gnathopod 2. However, this new species differs from *P. barnardi* in the shape of the posterior margin of male pereopod 5 basis, which has a very weak distal extension (well-developed in *P. barnardi*), biarticulated uropod 3 with the terminal article being vestigial (uniarticulated in *P. barnardi*) (Kudryashov and Tzvetkova 1975; Conlan 1983).

###### Podoceropsis pseudoclavapes sp. nov.

Taxon classificationAnimaliaAmphipodaPhotidae

22911750-328F-5579-8470-F98E2BA56DC9

http://zoobank.org/C3203587-E4C4-4E52-9EE2-744B2C4818A3

**Figure 24:**
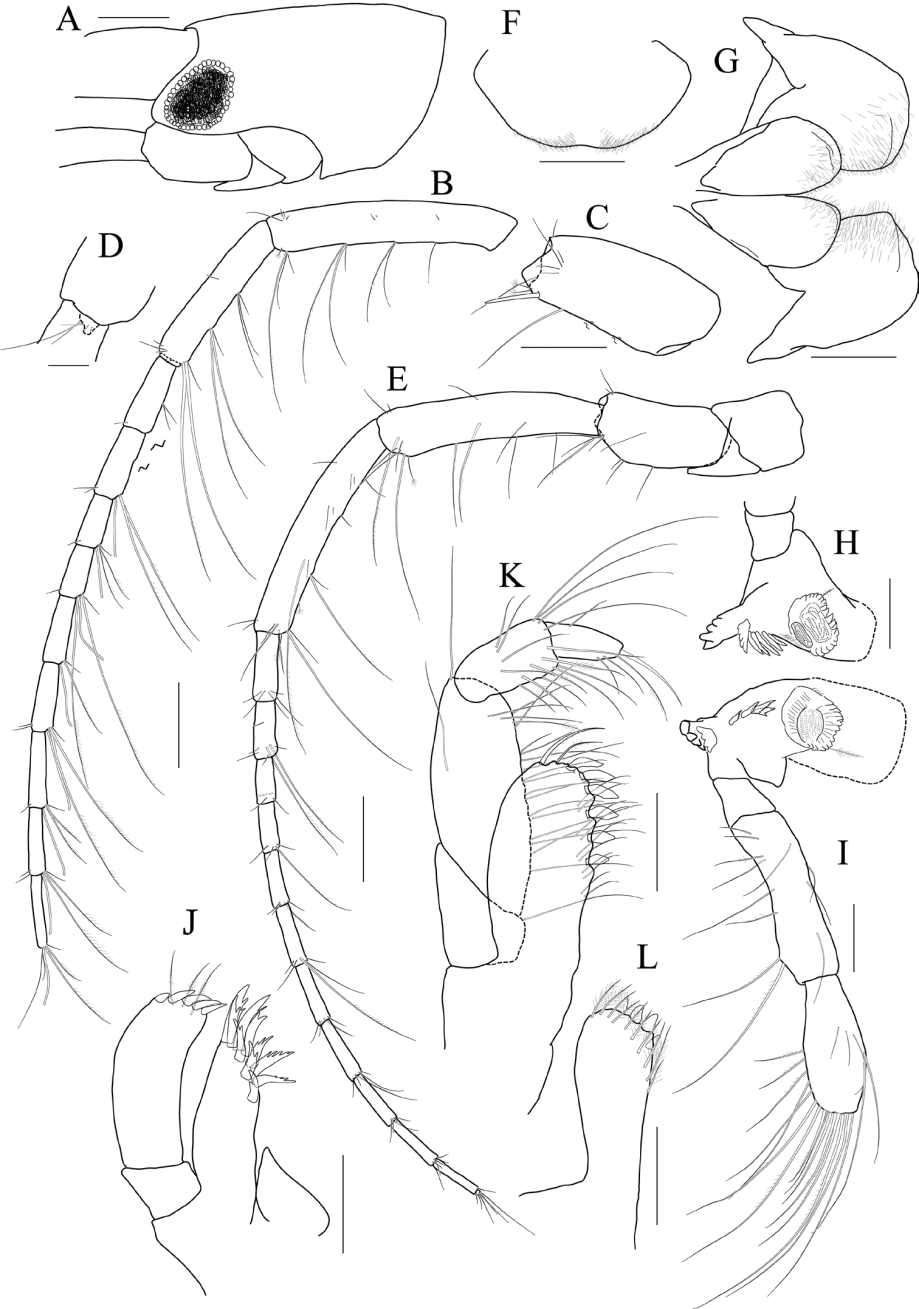
*Podoceropsis pseudoclavapes* sp. nov., holotype, NIBRIV0000806530, male, 3.2 mm, Bigin-do Island, South Korea. **A** head **B–D** antenna 1 **E** antenna 2 **F** upper lip **G** lower lip **H** right mandible **I** left mandible **J** maxilla 1 **K, L** maxilliped. Scale bars: 0.02 mm (**D**), 0.05 mm (**F–L**), 0.1 mm (**A–C, E**).

**Figure 25:**
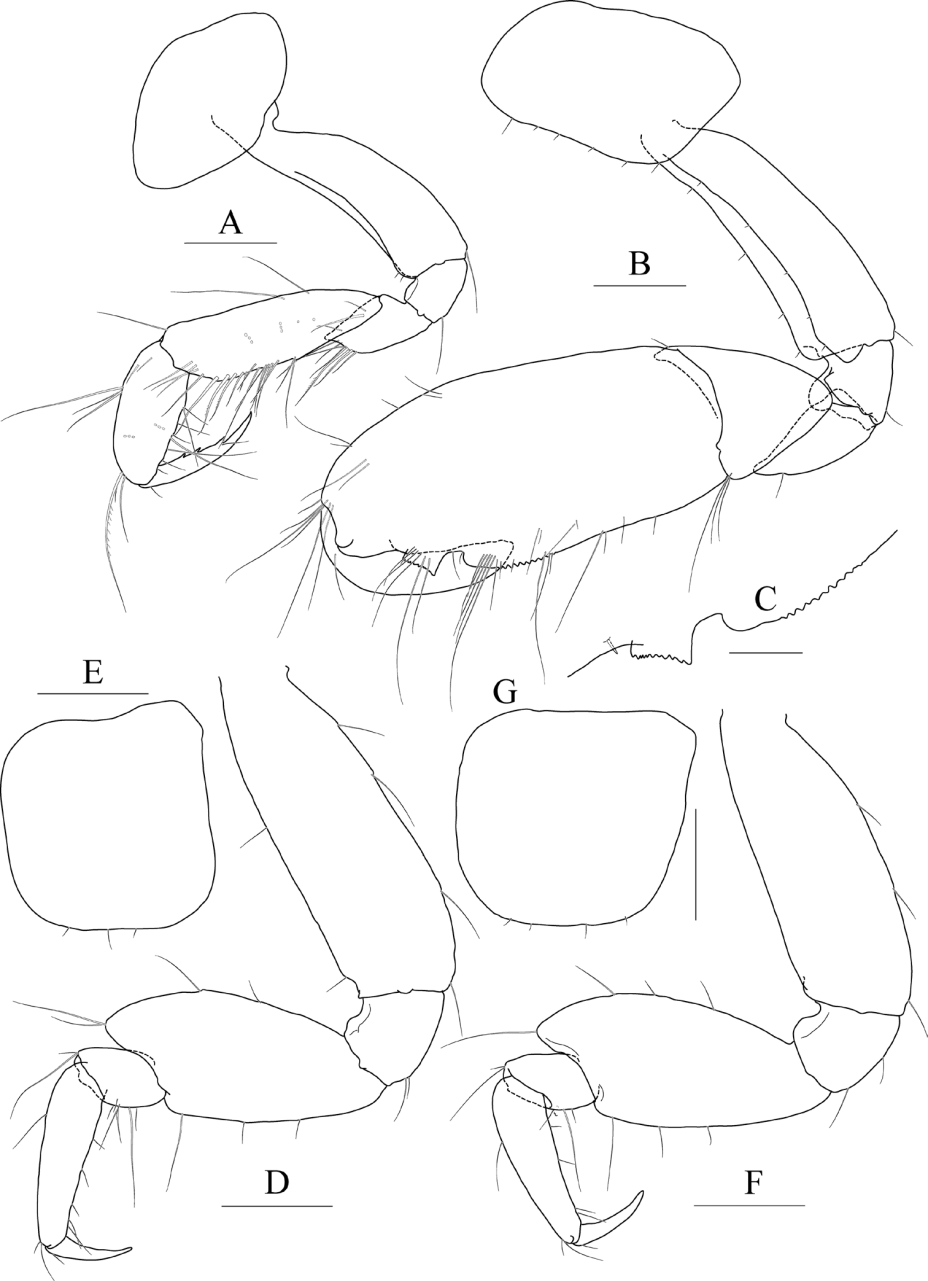
*Podoceropsis pseudoclavapes* sp. nov., holotype, NIBRIV0000806530, male, 3.2 mm, Bigin-do Island, South Korea. **A** gnathopod 1 **B** gnathopod 2 **C** palmer margin of gnathopod 2 **D** coxa 3 **E** pereopod 3 **F** coxa 4 **G** pereopod 4. Scale bars: 0.01 mm (**C**) 0.1 mm (**A, B, D–G**).

**Figure 26:**
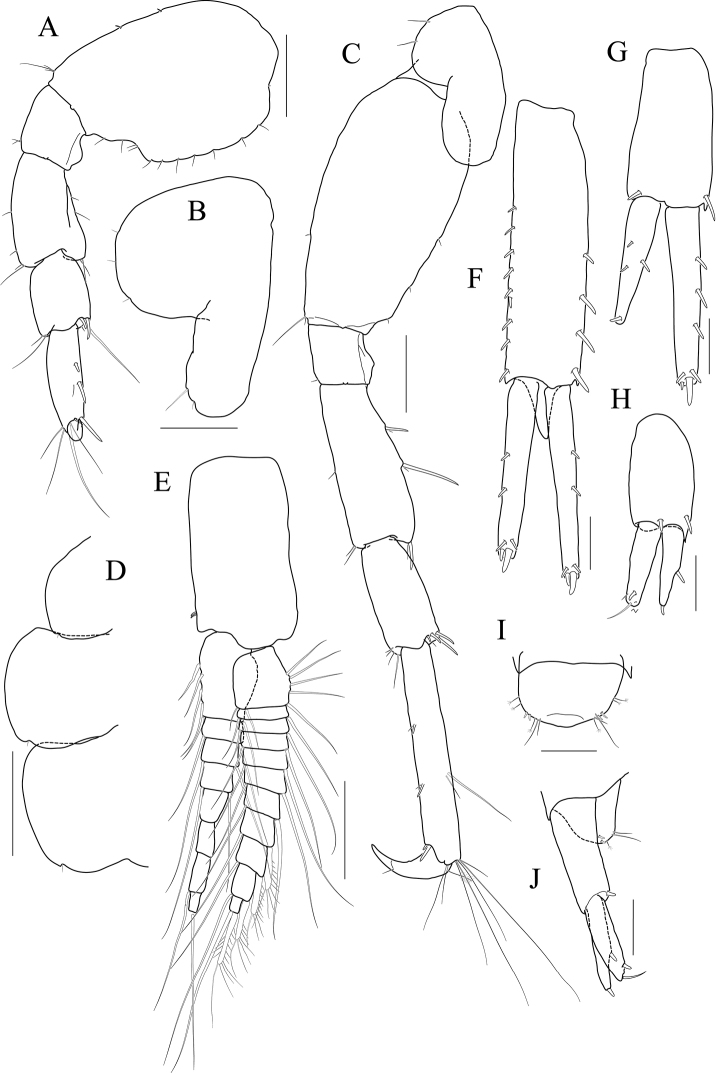
*Podoceropsis pseudoclavapes* sp. nov., holotype, NIBRIV0000806530, male, 3.2 mm, Bigin-do Island, South Korea. **A** coxa 5 **B** pereopod 5 **C** pereopod 6 **D** pleonal epimera **E** pleopod 2 **F** uropod 1 **G** uropod 2 **H** uropod 3 **I** telson **J** uropod 3 and telson, lateral. Scale bars: 0.05 mm (**F–J**), 0.1 mm (**A–C, E**), 0.2 mm (**D**).

**Figure 27:**
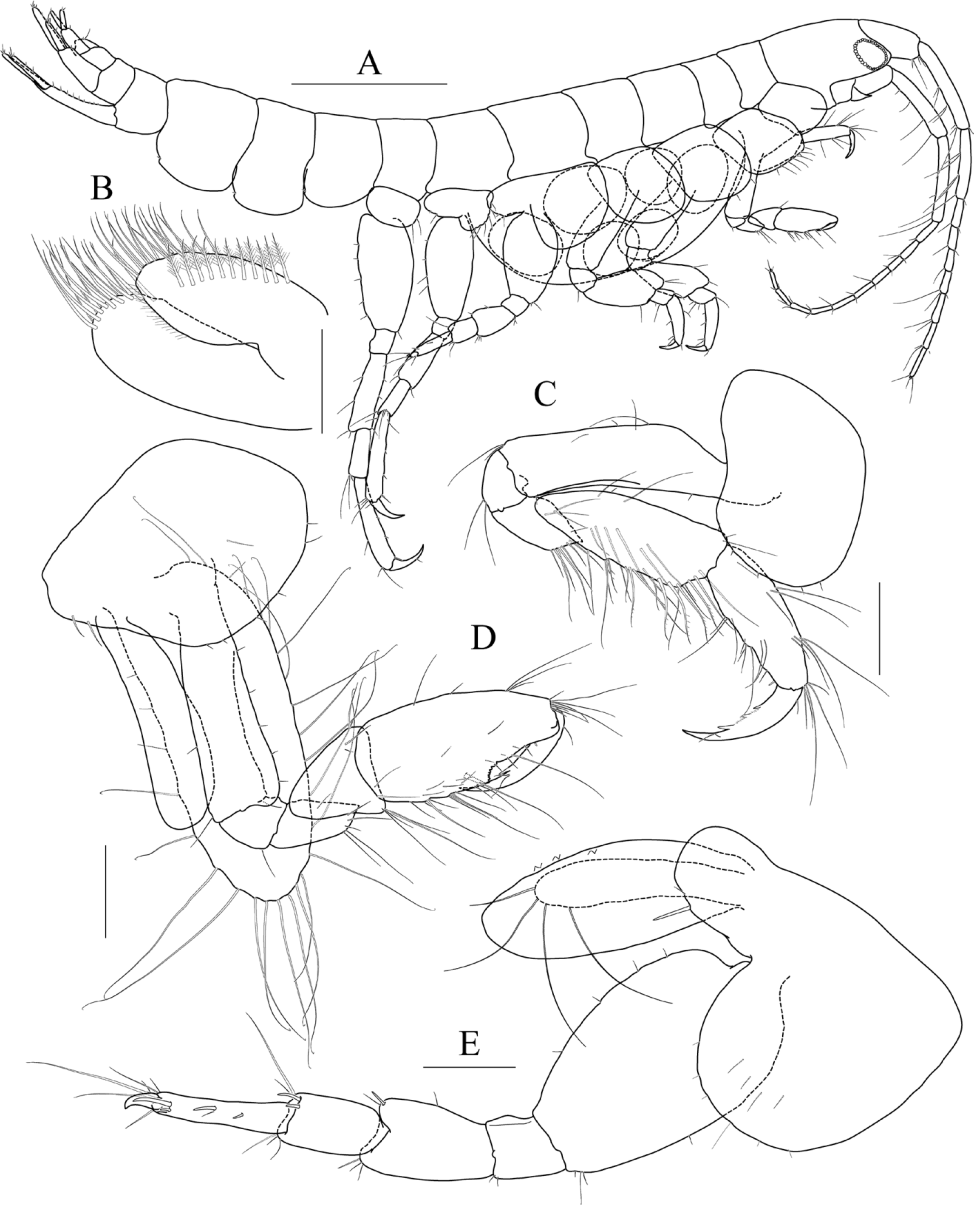
*Podoceropsis pseudoclavapes* sp. nov., paratype, NIBRIV0000807157, female, 2.5 mm, Bigin-do Island, South Korea. **A** habitus **B** maxilla 2 **C** gnathopod 1 **D** gnathopod 2 **E** pereopod 5. Scale bars: 0.05 mm (**B**), 0.5 mm (**A, C–E**).

Figs 24 , 25 , 26 , 27

####### Etymology.

The composite epithet of the specific name, *pseudoclavapes*, is a combination of the Greek word *pseudos*, and the specific name of *Podoceropsis clavapes* Jung, Choi, Kim & Yoon, 2017. This name refers to the similarity of this new species to *P. clavapes*.

####### Material examined.

Holotype: ♂ (3.2 mm), NIBRIV0000806530. Bigin-do Island, Gyeongsangnam-do, South Korea (34°42'52"N, 128°27'04"E), 18 Oct 2014, Scuba diving (about 20 m depth), by TW Jung. Paratypes: 1 ♂ (2.0 mm) and 3 ♀♀ (ovigerous; 2.1–2.7 mm), NIBRIV0000807157. Same data as holotype.

####### Diagnosis.

Gnathopod 1 carpus elongate, 0.9× as long as basis; dactylus as long as propodus. Gnathopod 2 propodus extremely stout, elongate ovoid, 1.2× as long as basis, half as wide as long, posterior margin with short undulate border distally, palm defined by rounded protrusion, with excavation successively posteriodistally and subquadrate protrusion bearing minutely serrated margin near dactyl base; dactylus stout, 0.4× as long as propodus, falcate, but apex quite obtuse. Pereopods 3 and 4 merus expanded, half as wide as long. Pereopod 5 basis posterior margin sinuated distally. Pereopod 5 basis posterior margin with additional lobe, but not produced distally. Uropod 3 rami subequal in length, 0.8× as long as peduncle.

####### Description.

***Holotype male.*** Head (Fig. 24A) lateral cephalic lobe subtriangular; eye subcircular, large, occupying most of lateral cephalic lobe; antennal sinus deep.

Antenna 1 (Fig. 24B–D) peduncle 1^st^ article stout, with one robust seta at posteriodistal corner; 2^nd^ article 1.3× as long as 1^st^ article; 3^rd^ article 0.7× as long as 2^nd^ article; accessory flagellum uniarticulated, vestigial; flagellum as long as peduncle 1^st^–3^rd^ articles combined, composed of ten articles (terminal article rudimentary).

Antenna 2 (Fig. 24E) 1.1× as long as antenna 1; peduncle 4^th^, 5^th^ articles 0.9× as long as 2^nd^ article of antenna; flagellum as long as peduncle 3^rd^–5^th^ articles combined, composed of twelve articles (terminal article rudimentary).

Upper lip (Fig. 24F) convex anteriorly, with notch in the middle, covered with minute setae.

Lower lip (Fig. 24G) inner lobe subovoid, outer lobe apex rounded, covered with minute setae; mandibular process well developed.

Mandibles (Fig. 24H, I) with 4-dentate incisor, 4-dentate lacinia mobilis, and four raker setae on left mandible; with 5-dentate incisor, 5-dentate lacinia mobilis, and five raker setae on right mandible; molar well developed, triturative; palp asymmetrical, composed of three articles, 3^rd^ article distally rounded, 0.8× as long as 2^nd^ article, with setae extending along most of posteriodistal margin.

Maxilla 1 (Fig. 24J) inner lobe small, produced distally, without setae; outer lobe with ten dentate robust setae on apical margin; palp biarticulated, distal article curved, a little swollen, with five setae on apical margin.

Maxilliped (Fig. 24K, L) inner lobe subrectangular, expanded distally, with three nodular setae apically and one medial nodular seta subdistally; outer lobe reaching half of palp 2^nd^ article, lined with eight robust setae along apex to medial margin; palp composed of four articles, 3^rd^ article slightly expanded distally, 0.4× as long as 2^nd^ article, 4^th^ article 0.8× as long as 3^rd^ article, with elongate seta apically (1.1× as long as 4^th^ article).

Gnathopod 1 (Fig. 25A) coxa rhomboid, produced anterioventrally, as long as wide, without setae on ventral margin; basis subtrapezoidal, 1.8× as long as coxa, scarcely setose, anterior margin lateral border lobate distally, posterior margin rather convex; carpus elongate, 0.9× as long as basis, 0.3× as wide as long, weakly widened distally, carpal lobe not developed; propodus 0.6× as long as carpus, rounded anteriorly, palm indistinct; dactylus elongate, as long as propodus, with three teeth on inner margin.

Gnathopod 2 (Fig. 25B, C) extremely stout, coxa subrhomboid, 1.4× as wide as long, convex anterioventrally, ventral margin slightly sinuous, with six setae; basis subtrapezoidal, 2.2× as long as coxa, anterior margin both lateral and medial borders forming lobes distally, but that of lateral obliquely truncated and more produced distally; ischium anterior margin with both lateral and medial lobes, medial lobe more developed; carpal lobe weakly developed; propodus extremely stout, elongate ovoid, 1.2× as long as basis, half as wide as long, anterior margin convex, slightly widened in proximal 2/3 margin, remaining distal 1/3 margin and dactylus forming smooth ellipse together, posterior margin with short undulate border distally, palm defined by rounded protrusion, with excavation successively posteriodistally and subquadrate protrusion bearing minutely serrated margin near dactylus base; dactylus stout, 0.4× as long as propodus, falcate, but apex quite obtuse.

Pereopod 3 (Fig. 25D, E) coxa subquadrate, with rounded corners; basis subtrapezoidal, somewhat expanded posteriorly, 0.4× as wide as long; merus as wide as basis, anterior margin extremely expanded, produced distal corner exceeding half of carpus; carpus short, 0.2× as long as basis; propodus diminished distally, half as long as basis; dactylus half as long as propodus.

Pereopod 4 (Fig. 25F, G) coxa 1.2× wider proximally than that of pereopod 3; other articles subequal to those of pereopod 3.

Pereopod 5 (Fig. 26A, B) coxa bilobed, anterior lobe rounded and expanded ventrally, 0.9× as wide as basis, posterior lobe narrow, extended backwards, with one robust and one simple seta; basis subovoid, 0.6× as wide as long, anterior margin more swollen proximally, posterior margin crenulated, lined with simple setae only, sinuated (notched) distally; merus widened distally, twisted, half as long as basis; carpus subrectangular, 0.7× as wide as long; propodus slightly plump, 1.3× as long as carpus, a pair of distal locking setae unequal in length (longer seta exceeding dactylus); dactylus falcate.

Pereopod 6 (Fig. 26C) 1.5× as long as pereopod 5; coxa bilobed, anterior lobe expanded ventrally, smaller than that of pereopod 5, with two simple setae anteriorly, posterior lobe slightly larger than anterior lobe, weakly dilated posterioventrally; basis subovoid, half as wide as long, with simple setae only, anterior margin convex, posterior margin scarcely crenulated, slightly expanded but flatter than anterior margin, bearing small notch distally; merus 0.6× as long as basis, 0.4× as wide as long, posterior margin expanded distally, with elongate robust setae (longest seta 0.3× as long as merus); carpus subrectangular, slightly widened distally, 0.7× as long as merus, half as wide as long; propodus linear, 2.0× as long as carpus, with a pair of unequal locking setae; dactylus stout, falcate, 0.3× as long as propodus.

Epimera 1–3 (Fig. 26D) each with a notch bearing minute seta on posterioventral corner, convex ventrally.

Uropod 1 (Fig. 26F) peduncle with distoventral spine 0.2× as long as peduncle, with four dorsomedial and nine dorsolateral robust setae; inner ramus 0.7× as long as peduncle, with two dorsomedial robust setae and one group of four robust setae on apex; outer ramus 0.9× as long as inner ramus, with two dorsolateral robust setae and one group of four robust setae on apex.

Uropod 2 (Fig. 26G) 0.7× as long as uropod 1; peduncle 0.6× as long as that of uropod 1, both dorsal margins with distal robust seta only; inner ramus 1.2× as long as peduncle, with 3 dorsomedial robust setae and one group of four robust setae on apex; outer ramus 0.7× as long as inner ramus, diminished distally, terminated by subacute-end bearing one robust seta.

Uropod 3 (Fig. 26H) 0.4× as long as uropod 1; peduncle 0.8× as long as that of uropod 2; inner ramus 0.8× as long as peduncle, diminished distally, with one robust seta on dorsomedial margin, terminated by one robust seta; outer ramus as long as inner ramus.

Telson (Fig. 26I, J) subtrapezoidal in dorsal view, slightly produced in apex, margins rounded, with a pair of robust setae on each side.

***Paratype female.*** Maxilla 2 (Fig. 27B) inner lobe with an oblique row of plumose setae on surface; outer lobe slightly longer than inner lobe.

Gnathopod 1 (Fig. 27C) not sexually dimorphic between both sexes.

Gnathopod 2 (Fig. 27D) as long as but stouter than female gnathopod 1, coxa subrhomboid, slightly produced anterioventrally, as long as wide, evenly rounded ventrally, oostegite broad, 1.3× as long as basis; basis anterior margin lateral borders forming lobe distally, but less produced distally than that of male; ischium with small anterior lobe only; merus rectangular, 0.4× as long as basis; carpus stout, anterior margin convex, carpal lobe weak; propodus stout, slightly elongate, 0.8× as long as basis, half as wide as long, palmar obliquely excavated, margin irregularly wrinkly, bearing one blunt spine medially,; dactylus half as long as propodus, inner margin minutely serrated, with one tooth.

Pereopod 5 (Fig. 27E) basis posterior margin not sinuated and without excavation distally.

####### Remarks.

*Podoceropsis clavapes* Jung, Choi, Kim & Yoon, 2017, which is the only known *Podoceropsis* species from Korean waters, is characterized by its peculiarly shaped gnathopod 2 in mature males: the propodus is markedly stout and enlarged (1.4 times as long as basis), the palmar margin has one proximal cavity and two distal protrusions, and the dactylus is stout and 0.8 times as long as the propodus (Jung et al. 2017). *Podoceropsis pseudoclavapes* sp. nov. has a similar shape of gnathopod 2 propodus in males to that of *P. clavapes*, but notably differs by gnathopod 1 propodus, which is half as long as the carpus (0.8 times as long as carpus in *P. clavapes*), the palmar margin of gnathopod 2, which has one rectangular protrusion and one distal cavity (one proximal cavity and two distal protrusions in *P. clavapes*), gnathopod 2 dactylus, which is 0.4 times as long as the propodus (0.8 times as long as propodus in *P. clavapes*), the merus of pereopods 3 and 4, which are half as wide as long (0.4 times as wide as long in *P. clavapes*), the posterior margin of pereopod 5 basis, which has an additional lobe, but it is not produced distally (also with lobes but differently produced distally in *P. clavapes*), and both rami of uropod 3, which are subequal in length and 0.9 times as long as the peduncle (inner ramus is 0.9 times as long as outer ramus and 0.7 times as long as peduncle in *P. clavapes*) (Jung et al. 2017).

*Podoceropsis angustimana* Conlan, 1983 also has a markedly stout and enlarged propodus and a similar excavation of the palmar margin of male gnathopod 2, but *P. pseudoclavapes* sp. nov. can be distinguished by the following differences: the propodus is half as long as the carpus (exceeding half the length in *P. angustimana*); pereopods 3 and 4 are widened; the posterior margin of pereopod 5 basis is lobed but not distally produced (posterior margin is slightly oblique and distally produced in *P. angustimana*); and uropod 3 rami are shorter than the peduncle (1.3 times as long as peduncle in *P. angustimana*) (Conlan 1983).

##### Key to known photid amphipods from Korean waters

**Table:** 

1	Uropod 3 with inner ramus notably shorter than outer ramus	**2**
–	Uropod 3 both rami subequal in length	**6**
2	Pereopods 5–7 elongate, slender; basis not expanded	***Exiliphotis petila* sp. nov.**
–	Pereopods 5–7 not elongate; basis moderately expanded	**3**
3	Females with stridulated ridges on gnathopod 2 basis and pereopod 3 coxa	***Photis stridulus* Jung, Choi, Kim & Yoon, 2017**
–	Females without stridulated ridges	**4**
4	Male gnathopod 1 notably longer than gnathopod 2; carpus very elongate, 0.9× as long as basis	***Photis longicarpus* sp. nov.**
–	Male gnathopod 1 shorter than gnathopod 2; carpus not elongate	**5**
5	Gnathopod 2 ischium posterior margin without lateral lobe; propodus palmar margin with one quadrate tooth medially, distal spine very weak	***Photis bronca* sp. nov.**
–	Gnathopod 2 ischium posterior margin forming lateral lobe produced distally; propodus palmar margin without quadrate teeth, with strong distal spine	***Photis posterolobus* sp. nov.**
6	Antenna 1 accessory flagellum uniarticulated, rudimentary	**7**
–	Antenna 1 accessory flagellum multiarticulated	**9**
7	Male gnathopod 2 propodus moderately stout, palm distinct. Uropod 2 outer ramus distally blunt with 4 robust setae	***Podoceropsis insinuomanus* sp. nov.**
–	Male gnathopod 2 propodus strongly elongate, palm indistinct. Uropod 2 outer ramus narrowing distally, with one robust seta	**8**
8	Male gnathopod 2 dactylus massive, 0.8× as long as propodus	***Podoceropsis clavapes* Jung, Choi, Kim & Yoon, 2017**
–	Male gnathopod 2 dactylus 0.4× as long as propodus	***Podoceropsis pseudoclavapes* sp. nov.**
9	Uropod 3 inner ramus narrowing distally, with one single small robust seta inserted at its tip; outer ramus distally blunt with a small second article	***Latigammaropsis careocavata* sp. nov.**
–	Uropod 3 both rami narrowing distally; outer ramus consisting of only one article	**10**
10	Male gnathopod 2 propodus very elongate, 1.9× as long as carpus. Male pereopod 7 basis moderately expanded	***Gammaropsis longipropodi* Hirayama, 1984**
–	Male gnathopod 2 propodus not elongate, 0.8× as long as carpus. Male pereopod 7 basis posterior margin notably expanded, with one strong notch posteriorly	***Gammaropsis utinomii* (Nagata, 1961)**

## Supplementary Material

XML Treatment for Exiliphotis

XML Treatment for Exiliphotis petila

XML Treatment for Latigammaropsis careocavata

XML Treatment for Photis bronca

XML Treatment for Photis longicaudata

XML Treatment for Photis longicarpus

XML Treatment for Photis posterolobus

XML Treatment for Podoceropsis insinuomanus

XML Treatment for Podoceropsis pseudoclavapes
